# SNORA56-mediated pseudouridylation of 28 S rRNA inhibits ferroptosis and promotes colorectal cancer proliferation by enhancing GCLC translation

**DOI:** 10.1186/s13046-023-02906-8

**Published:** 2023-12-05

**Authors:** Chang Xu, Zhixuan Bian, Xinyue Wang, Na Niu, Li Liu, Yixuan Xiao, Jiabei Zhu, Nan Huang, Yue Zhang, Yan Chen, Qi Wu, Fenyong Sun, Xiaoli Zhu, Qiuhui Pan

**Affiliations:** 1 Department of Laboratory Medicine, Shanghai Tenth People’s Hospital of Tongji University, Shanghai, 200072 China; 2 Department of Laboratory Medicine, Shanghai Children’s Medical Center, School of Medicine, Shanghai Jiao Tong University, Shanghai, 200127 China; 3 College of Health Science and Technology, School of Medicine, Shanghai Jiao Tong University, Shanghai, 200025 China; 4 Shanghai Key Laboratory of Clinical Molecular Diagnostics for Paediatrics, Shanghai, 200127 China; 5 Sanya Women and Children’s Hospital Managed by Shanghai Children’s Medical Center, Sanya, 572000 China; 6 Department of Central Laboratory, Shanghai Tenth People’s Hospital of Tongji University, Shanghai, 200072 China

**Keywords:** Colorectal cancer, SNORA56, Proliferation, Pseudouridylation, Ferroptosis, GCLC, Biomarker, Therapy

## Abstract

**Background:**

Colorectal cancer (CRC) is one of the most common malignancies and is characterized by reprogrammed metabolism. Ferroptosis, a programmed cell death dependent on iron, has emerged as a promising strategy for CRC treatment. Although small nucleolar RNAs are extensively involved in carcinogenesis, it is unclear if they regulate ferroptosis during CRC pathogenesis.

**Methods:**

The dysregulated snoRNAs were identified using published sequencing data of CRC tissues. The expression of the candidate snoRNAs, host gene and target gene were assessed by real-time quantitative PCR (RT-qPCR), fluorescence in situ hybridization (FISH), immunohistochemistry (IHC) and western blots. The biological function of critical molecules was investigated using in vitro and in vivo strategies including Cell Counting Kit-8 (CCK8), colony formation assay, flow cytometry, Fe^2+^/Fe^3+^, GSH/GSSG and the xenograft mice models. The ribosomal activities were determined by polysome profiling and O-propargyl-puromycin (OP-Puro) assay. The proteomics was conducted to clarify the downstream targets and the underlying mechanisms were validated by IHC, Pearson correlation analysis, protein stability and rescue assays. The clinical significance of the snoRNA was explored using the Cox proportional hazard model, receiver operating characteristic (ROC) and survival analysis.

**Results:**

Here, we investigated the SNORA56, which was elevated in CRC tissues and plasma, and correlated with CRC prognosis. SNORA56 deficiency in CRC impaired proliferation and triggered ferroptosis, resulting in reduced tumorigenesis. Mechanistically, SNORA56 mediated the pseudouridylation of 28 S rRNA at the U1664 site and promoted the translation of the catalytic subunit of glutamate cysteine ligase (GCLC), an indispensable rate-limiting enzyme in the biosynthesis of glutathione, which can inhibit ferroptosis by suppressing lipid peroxidation.

**Conclusions:**

Therefore, the SNORA56/28S rRNA/GCLC axis stimulates CRC progression by inhibiting the accumulation of cellular peroxides, and it may provide biomarker and therapeutic applications in CRC.

**Supplementary Information:**

The online version contains supplementary material available at 10.1186/s13046-023-02906-8.

## Background

Colorectal cancer (CRC) is the second leading cause of cancer deaths worldwide, and its incidence and mortality are increasing [1]. CRC diagnosis relies on imaging and the detection of plasma biomarkers, such as carcinoembryonic antigen (CEA) and carbohydrate antigen199 (CA199), but these methods have limited sensitivity and specificity [2]. Histological analysis, which is used for pathological staging and to guide subsequent management, is not suitable for extensive CRC screening because of its invasiveness. Consequently, CRC patients with poor prognosis result from the lack of effective therapeutic targets and accurate biomarkers for early detection. Currently, CRC is mainly treated through surgery combined with chemotherapy, radiotherapy, and immunotherapy. However, CRC has a high risk of metastasis and recurrence because of drug resistance [3]. Thus, a better understanding of the mechanisms that underlie CRC progression is needed for the development of biomarkers for early diagnosis, as well as effective therapies.

Small nucleolar RNAs (snoRNAs), a family of conserved non-coding RNAs with a length of 60–300 nucleotides, are mainly derived from the introns of host genes. Based on their structural elements, snoRNAs are classified as box C/D snoRNAs or box H/ACA snoRNAs, which interact with evolutionarily conserved ribonucleoproteins to modulate the processing of ribosomal RNAs (rRNAs) through 2’-O-methylation and pseudouridylation, respectively, thereby regulating ribosome subunit maturation and translation [4]. Recent studies have shown that snoRNAs are frequently dysregulated in various cancers and that they contribute to tumorigenesis and cancer progression through diverse mechnisms [5–7]. This implies that snoRNAs might have therapeutic value. For example, SNORA38B plays an oncogenic role in non-small cell lung cancer by binding to E2F1 and regulating the GAB2/AKT/mTOR pathway to affect immunotherapy sensitivity [8]. Moreover, SNORD12C/78 functions in CRC pathogenesis by guiding 2’-O-Methylation at Gm3878 and Gm4593 sites, thereby increasing oncogene translation [9]. Notably, because snoRNAs are stable in body fluids [10], they have the potential for use as non-invasive biomarkers for early CRC detection and prognosis prediction.

The finding that CRC initiation and progression is accompanied by flexible metabolic reprogramming may lead to the identification of metabolic therapeutic targets and have an impact on treatment responses [11]. Moreover, the metabolic adaptation involving ferroptosis has been proposed as a potential therapeutic target in CRC [12]. Ferroptosis, as a programmed cell death, is mainly regulated by system Xc^−^, which imports cystine for the synthesis of reduced glutathione (GSH). Through the catalytic action of glutathione peroxidase 4 (GPX4), GSH suppresses the formation of lipid peroxides and inhibits ferroptosis. GCLC, the rate-limiting enzyme in GSH synthesis, suppresses ferroptosis by catalyzing the ligation of cysteine to glutamate, therefore promoting CRC metastasis [13]. GCLC is reported to maintain glutamate homeostasis during cystine starvation and to protect from ferroptosis in a glutathione-independent manner, implying that changes in GCLC function are crucial for ferroptosis resistance [14]. Past studies have revealed that high GCLC expression contributes to oxaliplatin detoxification in CRC peritoneal metastases derived organoids [15], highlighting the suppression of GCLC as a potential strategy for CRC treatment. Although GCLC expression is driven by the transcription factor, nuclear factor erythroid 2-related factor 2 during several pathogeneses [16, 17], the translational regulation of GCLC during CRC is not fully understood. Notably, recent studies indicate that non-coding RNAs regulate ferroptosis [18, 19]. Indeed, studies have identified ferroptosis-associated long non-coding RNA signatures for predicting the prognosis of various cancers [20–22]. However, the relationship between snoRNAs and ferroptosis is unclear.

This study investigated SNORA56, which is overexpressed in CRC tissues and cells. SNORA56, located in an intronic region of dyskerin 1 (DKC1), is 129 nucleotides long and correlated with CRC prognosis. We first used in vitro and in vivo strategies to confirm that SNORA56 promotes CRC proliferation while suppressing ferroptosis. We identify GCLC, the rate-limiting enzyme in GSH biosynthesis, as a major downstream target of SNORA56, which promotes GCLC translation by mediating the pseudouridylation of 28 S rRNA, thereby inhibiting ferroptosis and promoting CRC survival. These findings provide novel insights into the roles of SNORA56 in CRC.

## Methods

### Cell culture and clinical specimens

The human CRC cells, HCT8, HCT116, HT29, and LoVo were purchased from BeNa Culture Collection (Beijing, China), whereas SW480 and Caco2 (CRC cells), HEK-293T cells, and the human normal intestinal epithelial cell line, HIEC, were obtained from the cell bank of the Type Culture Collection of the Chinese Academy of Sciences (Shanghai, China). HEK-293T, SW480, and Caco2 cells were cultured in DMEM (Gibco, USA) supplemented with 10% fetal bovine serum (FBS, Gibco, USA) and 1% penicillin-streptomycin (PS, NCM Biotech, China). LoVo cells were cultured in F-12 K medium (Gibco, USA) supplemented with 10% FBS and 1% PS. HCT8, HCT116, HT29, and HIEC cells were cultured in RPMI-1640 (Gibco, USA) containing 10% FBS and 1% PS. All cell lines were cultured in a humified incubator, at 37 °C and 5% CO_2_.

CRC and paired para-cancerous tissues, as well as plasma samples, were collected at Shanghai Tenth People’s Hospital between 2020 and 2023. Ethical approval for the study was granted by the ethics committee of Shanghai Tenth People’s Hospital. Two pathologists confirmed CRC diagnosis and provided detailed clinicopathological information.

### Plasmids, antisense oligonucleotides (ASOs), and cell transfection

Human SNORA56 (NR_002984.1), GCLC (NM_001197115.2), and the full-length cDNAs for the SNORA56 mutants, MUT1 (mutated from AGUUAUCC to UCAAUAGG) and MUT2 (mutated from GGGAG to CCCUC) were synthesized by Ke Lei Biological Technology (Shanghai, China) and cloned into the pCDNA3.1 vector. The SNORA56 cDNA was also cloned into the pLVX-AcGFP vector for simultaneous expression and the product named LV-SNORA56. SNORA56-targeting sgRNAs (sgSNORA56) were designed using CRISPOR [23] and cloned into the lentiCRISPRv2 plasmid (sgNC). ASOs were purchased from RIBOBIO corporation (Guangzhou, China). The plasmids and ASOs were transiently transfected using Lipofectamine 2000 (Invitrogen, USA) according to manufacturer instructions. For stable overexpression or knockdown, packaged plasmids (psPAX2 and pMD2.G) and the target plasmid were co-transfected into HEK-293T cells followed by lentivirus harvesting at 24, 48, and 72 h. The indicated cell lines were then incubated overnight with the lentivirus and polybrene (Santa Cruz, USA). Stably transfected cells were selected through continuous treatment (two weeks) with puromycin (Invitrogen, USA) at 2 µg/ml.

### Quantitative reverse transcription PCR (RT-qPCR)

Total RNA was isolated from cells or plasma using Trizol (Invitrogen, USA). RNA was extracted from CRC tissues using a FastPure Cell/Tissue Total RNA Isolation kit V2 (Vazyme, Nanjing) following the manufacturer’s protocol. RNA was retrotranscribed using a PrimeScript^™^ RT reagent Kit with or without gDNA Eraser (RR037A, RR047A, TaKaRa, Japan). RT-qPCR analysis was done using TB Green^®^ Premix Ex Taq^™^ II (RR820A, TaKaRa, Japan) on a QuantStudio Dx Real-Time PCR system (Thermo, USA) using U6 to normalize snoRNAs expression and 18 S as the reference gene for all other genes. Relative RNA levels were determined using the 2^-ΔΔCt^ method.

### Cell proliferation assays

Cell Counting Kit-8 (CCK8, Beyotime, China) was used to assess cell proliferation. Briefly, cells were seeded into 96-well plates at a density of 1,000 cells/well. At indicated time points, the medium was replaced with fresh medium containing 10% CCK8 reagent, and absorbance read at 450 nm on a SpectraMax iD5 multimode plate reader (Molecular Devices, USA) after a three-hour incubation. For colony formation analysis, cells were plated on 12-well plates at a density of 1,000 cells/per well and cultured for one to two weeks. They were then fixed with 4% paraformaldehyde (Biosharp, China) and stained with 1% crystal violet for visualization.

### Animal experiments

Four-week-old nude mice (male) were purchased from Charles River Corp (Beijing, China) and subcutaneously injected with 1 × 10^7^ HT29 sgNC or sgSNORA56 cells on either flank. Tumor growth was monitored every other day and tumor volume was calculated using the formula: ab^2^/2, where a = length and b = width). For the IKE sensitivity assay, 20 male mice aged four weeks were subcutaneously injected with HT29 sgNC or sg SNORA56 cells on either flank, and tumor volumes were monitored every two days. When the mean volume reached 90 mm [3], the mice were randomly divided into two subgroups and intraperitoneally injected with IKE (Selleck, USA) at 50 mg/kg, daily for 2 weeks. For injection, the IKE was dissolved in a solution of 65% D5W (5% dextrose in water, Biosharp, China), 5% Tween-80 (Selleck, USA) and 30% PEG-300 (Selleck, USA). Control mice were treated with the solvent only. The mice were euthanized at the end of the study, followed by tumor harvesting and tumor weight measurement.

### Western blot analyses

Proteins were extracted from cells by incubating them on ice for 10 min in RIPA buffer (Beyotime, China) supplemented with phosphatase and protease inhibitors (Sangon Biotech, Shanghai, China). The lysate was then cleared by centrifugation at 12,000 revolutions per minute, for 15 min, at 4 °C. Protein concentration was determined using a BCA protein assay kit (NCM Biotech, China). Proteins were then boiled in 6× loading buffer (Beyotime, China) for 10 min, and equal amounts resolved using SDS-PAGE. They were then transferred onto nitrocellulose membranes, blocked with 5% BSA in TBST, and then incubated with indicated primary antibodies (anti-GCLC and anti-GAPDH) at 4 °C, overnight. They were then washed thrice with TBST and incubated with secondary antibodies at room temperature for one hour. Finally, the protein signal was visualized on an Odyssey imaging system (LI-COR, USA).

### Immunohistochemistry (IHC) and fluorescent in situ hybridization (FISH)

Paraffin-embedded tissues or CRC tissue microarrays were used for IHC analysis. Briefly, following deparaffinization, hydration, antigen retrieval, and blocking, slides were incubated with antibodies against Ki67 or GCLC at 4 °C overnight. Next, the slides were incubated with biotinylated secondary antibody and peroxidase-labeled streptavidin, and the signal was developed using the diaminobenzidine chromogenic substrate. For FISH analysis, a SNORA56-specific digoxin-labeled probe was synthesized by Biosune Biotech Corp (Shanghai, China). The FISH experiments on CRC tissue microarray and panoramic scanning were done by Runnerbio Corp (Shanghai, China). The staining intensity were independently determined by two blinded pathologists.

### 4D label-free qualitative proteomics

Proteins were extracted from the HCT8 sgNC and sgSNORA56 cells and label-free qualitative proteomics carried out by PTM Bio Corp (Shanghai, China). Briefly, the tryptic peptides dissolved in solvent A (0.1% formic acid and 2% acetonitrile in water) were directly loaded onto a homemade reversed-phase analytical column. The peptides were then separated in solvent B (0.1% formic acid in acetonitrile) at a constant flow rate, using a nanoElute UHPLC system (Bruker Daltonics). Next, the peptides were subjected to capillary electrophoresis, followed by mass spectrometry on a timsTOF Pro system (Bruker Daltonics), which was done in the parallel accumulation serial fragmentation mode. Data were processed using the MaxQuant search engine (v.1.6.15.0) and referenced against the human SwissProt database. FDR was adjusted to < 1%.

### Glutamate cysteine ligase (GCL) activity assay

Fresh cells (more than 10^7^) were ultrasonically lysed on ice. A GCL enzyme activity assay kit (BC1210, Solarbio, China) was then used to assess GCL activity according to manufacturer instructions via absorbance readings. The GCL activity was normalized through relative cell count.

### Measurements of iron concentration and the GSH/GSSG ratio

An iron assay kit (ab83366, Abcam, UK) was used to measure total iron, Fe^2+^, and Fe^3+^. Iron concentration was calculated using the following formula: iron concentration (µM) = iron content (nmol)/sample volume (µL) × dilution factor. GSH and GSSG assay kits (BC1175 and BC1180, respectively, Solarbio, China) were used to determine the GSH/GSSG ratios according to manufacturer guidelines.

### Flow cytometry

Lipid peroxidation and cell death were assessed as previously described [24]. Briefly, cells were collected and stained with BODIPY-C11 (Invitrogen, USA) at 37 °C avoiding light, for 30 min, followed by lipid peroxidation measurement using flow cytometry. For live cell analysis, cells were incubated with propidium iodide (Invitrogen, USA) for five min at room temperature avoiding light followed by flow cytometry.

### Polysome profiling

A total of 2 × 10^7^ cells in a lysis buffer (50 mM Tris-HCl pH7.4, 100 mM NaCl, 5 mM MgCl_2_, 100 µg cycloheximide, and 1% Triton X-100) supplemented with a protease inhibitor cocktail and an RNase inhibitor, were incubated on ice for 15 min. The supernatants were then collected by centrifuging at 12,000 revolutions per minute for 10 min, at 4 °C. The lysates were then loaded into 10–50% sucrose density gradients prepared using a BioComp Gradient Master Model 108 (BioComp, Canada) and then separated by ultracentrifugation using an SW41Ti rotor (Beckman Coulter, USA) at 23,000 g for three hours at 4 °C. The centrifuged solution was then divided into 13 equal fractions and their absorbances measured simultaneously at 260 nm, from top to bottom on an automatic separation and analysis system (BioComp, Canada).

### O-propargyl-puromycin (OP-Puro) assay

The incorporation of OP-Puro into nascent polypeptides indicates protein synthesis activity. Cells growing on six-well plates were treated with puromycin at 20 µg/mL for two hours and then harvested for protein extraction and western blot analysis using anti-puro antibody (Millipore, USA).

### Protein stability assay

GCLC protein stability was examined as described previously [6]. Briefly, SNORA56-silenced or SNORA56-overexpressing cells were treated with cycloheximide (CHX, Sigma, USA) at 100 µg/ml to block translation and then harvested at 0, 4, 8, 12, 24 h after treatment. Proteins were then extracted, followed by western blot analysis of GCLC level.

### Statistical analyses

All experiments were done in triplicate. Data analyses were done using SPSS version 25.0 (IBM, Germany) and GraphPad Prism version 9.0 (San Diego, USA). Variables with normal distribution were compared using the *t*-test for two groups, or one-way analysis of variance (ANOVA) for multiple groups. The Wilcoxon rank test was used to analyze paired data. Tumor growth curves were analyzed using two-way ANOVA (Bonferroni’s test). Correlation analysis was done using the Spearman rank test. Chi-square or Fisher’s exact test were used to compare clinical characters in SNORA56 high and low expression groups. Kaplan–Meier analysis was used for survival analysis and the survival differences were compared using the Log-rank test. The Cox proportional hazards model was used to analyze the impact of variables on survival. Receiver operating characteristic (ROC) curve analysis and area under curve (AUC) were used to assess diagnostic potential. Results are presented as the mean ± standard deviation. *P* < 0.05 indicates statistically significant differences. *, **, and *** indicate *P* < 0.05, < 0.01, and < 0.001, respectively.

## Results

### SNORA56 is highly expressed and correlated with poor prognosis in CRC

**Fig. 1:**
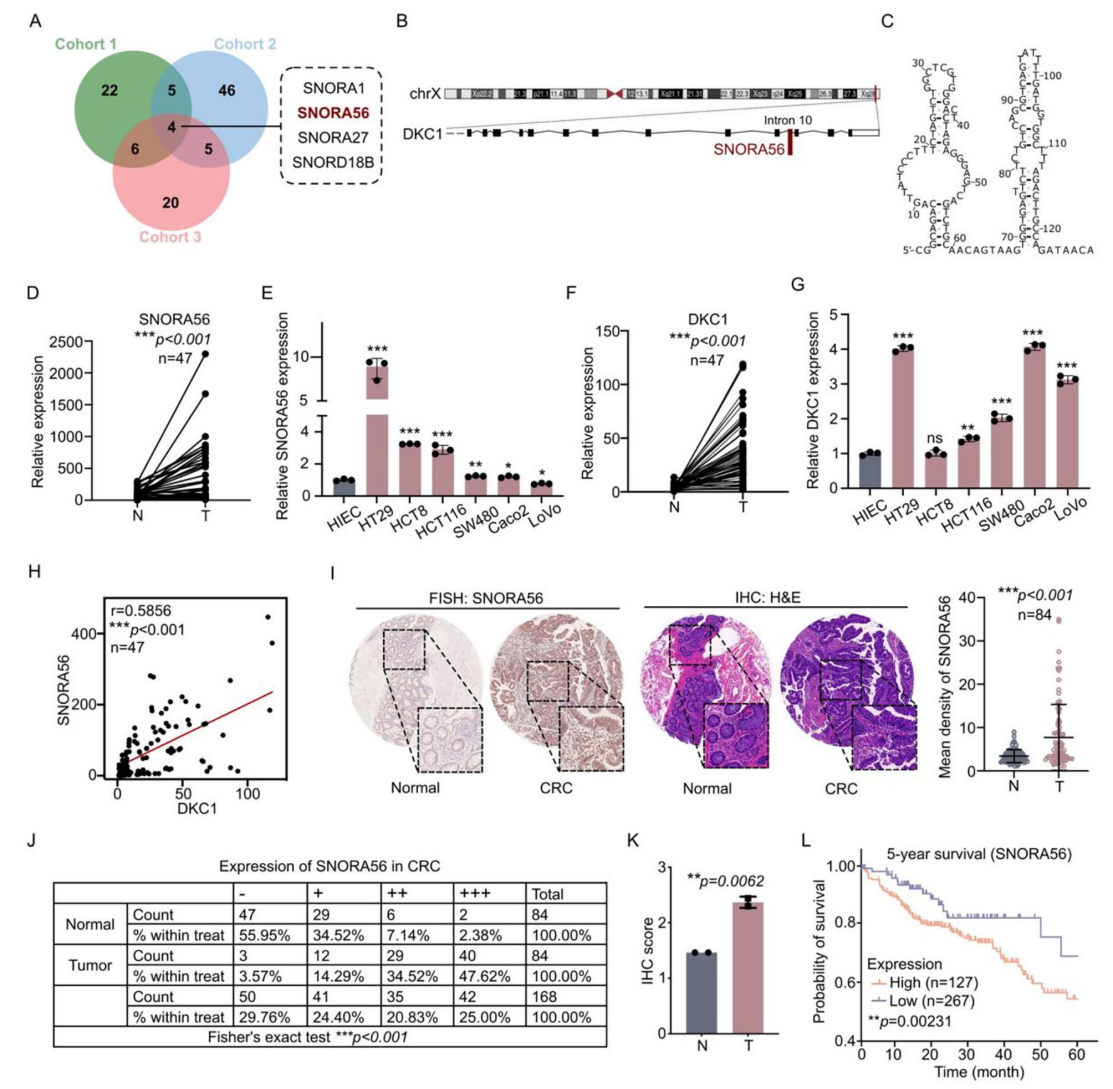
SNORA56 upregulation in CRC correlates with poor prognosis. **(A)** A Venn diagram of three published cohorts reporting upregulation of snoRNAs in CRC tissues. Cohort 1 was obtained from the published article [25]. Cohort 2 is from the Gene Expression Omnibus dataset GSE20916^26^. Cohort 3 is from our previously published work [29]. **(B)** Visualization of the genomic location of SNORA56 in its host gene, DKC1, on the UCSC Genome Browser. **(C)** A schematic representation of the structure of SNORA56. **(D, F)** The relative expression of SNORA56 and DKC1 in 47 paired CRC and adjacent non-tumor tissues was revealed using RT-qPCR. **(E, G)** The relative expression of SNORA56 and DKC1 in HIEC, HT29, HCT8, HCT116, SW480, Caco2, and LoVo cells was determined using RT-qPCR. **(H)** Analysis of the correlation between the expression of SNORA56 and DKC1. **(I)** SNORA56 FISH analysis and hematoxylin & eosin (H&E) staining in CRC tissue microarray. The mean density of SNORA56 signals was measured using Image Pro Plus. **(J–K)** SNORA56 staining intensity and the IHC score of CRC tissue microarray. **(L)** Kaplan Meier curve of the 5-year survival analysis in SNORA56 high and low groups using TCGA_COAD datasets from the SNORic database

To determine the differential expression of snoRNA in CRC, we performed snoRNA sequencing on five CRC and its adjacent non-tumor tissues in our previous work, then compared our data with two published snoRNA datasets from CRC tissues [25, 26]. This analysis identified the snoRNAs, SNORA1, SNORA56, SNORA27, and SNORD18B, as being differentially upregulated in all three datasets (Fig. 1A). Next, we used RT-qPCR to validate their expression in 47 paired CRC and corresponding adjacent non-tumor tissues. SNORA56, as the most upregulated, was selected for further investigation (Fig. 1D, S1A–C). SNORA56, which is derived from the tenth intron of the DKC1 gene, is 129 nucleotides long and contains the conserved H/ACA box (Fig. 1B–C). Pan-cancer analysis of The Cancer Genome Atlas (TCGA) dataset revealed that SNORA56 was most significantly enriched in CRC (Figure S2).

Next, we used RT-qPCR and FISH assay to validate SNORA56 upregulation in various CRC cell lines and CRC tissue microarrays, respectively. Our analyses revealed that SNORA56 levels in CRC cell lines (HT29, HCT8, HCT116, SW480 and Caco2, except for LoVo) were markedly higher than in the normal human intestinal epithelial cell line (HIEC) (Fig. 1E). Moreover, FISH revealed significantly higher SNORA56 levels in CRC tissues compared to the adjacent non-tumor tissues (Fig. 1I–K, S1D), suggesting that SNORA56 might influence CRC progression. Furthermore, analysis of the expression of DKC1, from which SNORA56 is derived, revealed that as with SNORA56, DKC1 mRNA levels were frequently upregulated in CRC tissues and cells (Fig. 1F–G). Moreover, in CRC tissues, there was a significant positive correlation between SNORA56 and DKC1 at the transcriptional level using Spearman rank correlation analysis (r = 0.5856, *P* < 0.001, Fig. 1H), implying that SNORA56 is co-transcribed with DKC1. Because DKC1 is proposed as a CRC prognostic biomarker [27], we evaluated the potential role of SNORA56 in CRC prognosis using TCGA_COAD data from SNORic [28]. This analysis found that CRC patients with higher SNORA56 levels had a poorer 5-year survival rate (Fig. 1L), indicating that SNORA56 is involved in CRC pathogenesis and highlighting SNORA56 as a potential biomarker for CRC prognosis.

### SNORA56 promotes CRC cell proliferation in vitro and in vivo

**Fig. 2:**
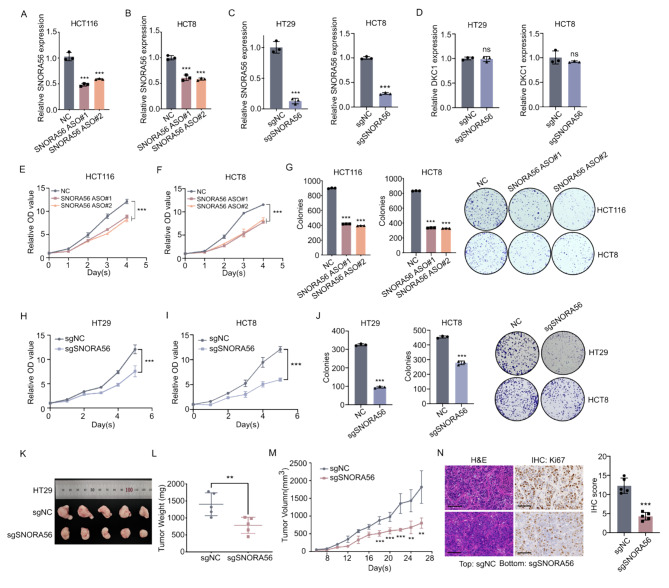
SNORA56 promotes CRC proliferation in vitro and in vivo. **(A–C)** The efficiency of SNORA56 silencing in CRC cell lines upon the indicated transfections was determined using RT-qPCR. **(D)** RT-qPCR analysis of DKC1 mRNA levels in HT29 cells stably transfected with sgNC and sgSNORA56. **(E–F, H–I)** CCK8 analysis of the proliferation of CRC cells upon the indicated transfections. **(G, J)** Colony formation analysis in CRC cells upon transient or stable SNORA56 knockdown vs. the negative control. **(K)** Tumors from nude mouse subcutaneous xenografts bearing HT29 sgNC cells (right) and sgSNORA56 cells (left). **(L–M)** Tumor weights and growth curves based on tumor volume measurements every two days. **(N)** IHC analyses of H&E and Ki67 in the xenograft tumors. Ki67 scores were calculated in HT29 sgNC and sgSNORA56 xenografts. Scale bar: 100 μm

To assess the biological function of SNORA56 in CRC, we transiently downregulated its expression using two independent antisense oligonucleotides (ASOs) in HCT116 and HCT8 cells. Moreover, we generated HIEC and CRC cell lines (HCT8 and HT29) in which SNORA56 was stably up- or down-regulated using lentiviral transduction of overexpressed plasmids (LV-NC and LV-SNORA56) or CRISPR/Cas9 system (sgNC and sgSNORA56), respectively. Transfection efficiency was assessed using RT-qPCR (Fig. 2A–C, S3A). Notably, stable SNORA56 overexpression or silencing did not affect DKC1 levels, suggesting that SNORA56 functions independently of DKC1 (Fig. 2D, S3B). Next, CCK-8 and colony formation analyses of the proliferative potential of CRC cells in vitro revealed that SNORA56 depletion disrupts CRC cell viability (Fig. 2E–J), whereas its upregulation markedly promotes proliferation (Figure S3C–D). Moreover, a xenograft nude mouse model of CRC using HT29 sgNC and sgSNORA56 cells was constructed through subcutaneous injection (Fig. 2K). Results revealed that the weights and volumes of tumors from the SNORA56-deficient cells (sgSNORA56) were markedly lower than in those from the control (sgNC) (Fig. 2L–M). Consistent with this observation, Ki67 levels, a canonical proliferative marker, in SNORA56 depleted xenografts were apparently decreased with the analysis of IHC, which confirmed that SNORA56 significantly promoted CRC cell proliferation in vivo (Fig. 2N).

### SNORA56 drives tumorigenesis by promoting 28 S rRNA maturation and translation

**Fig. 3:**
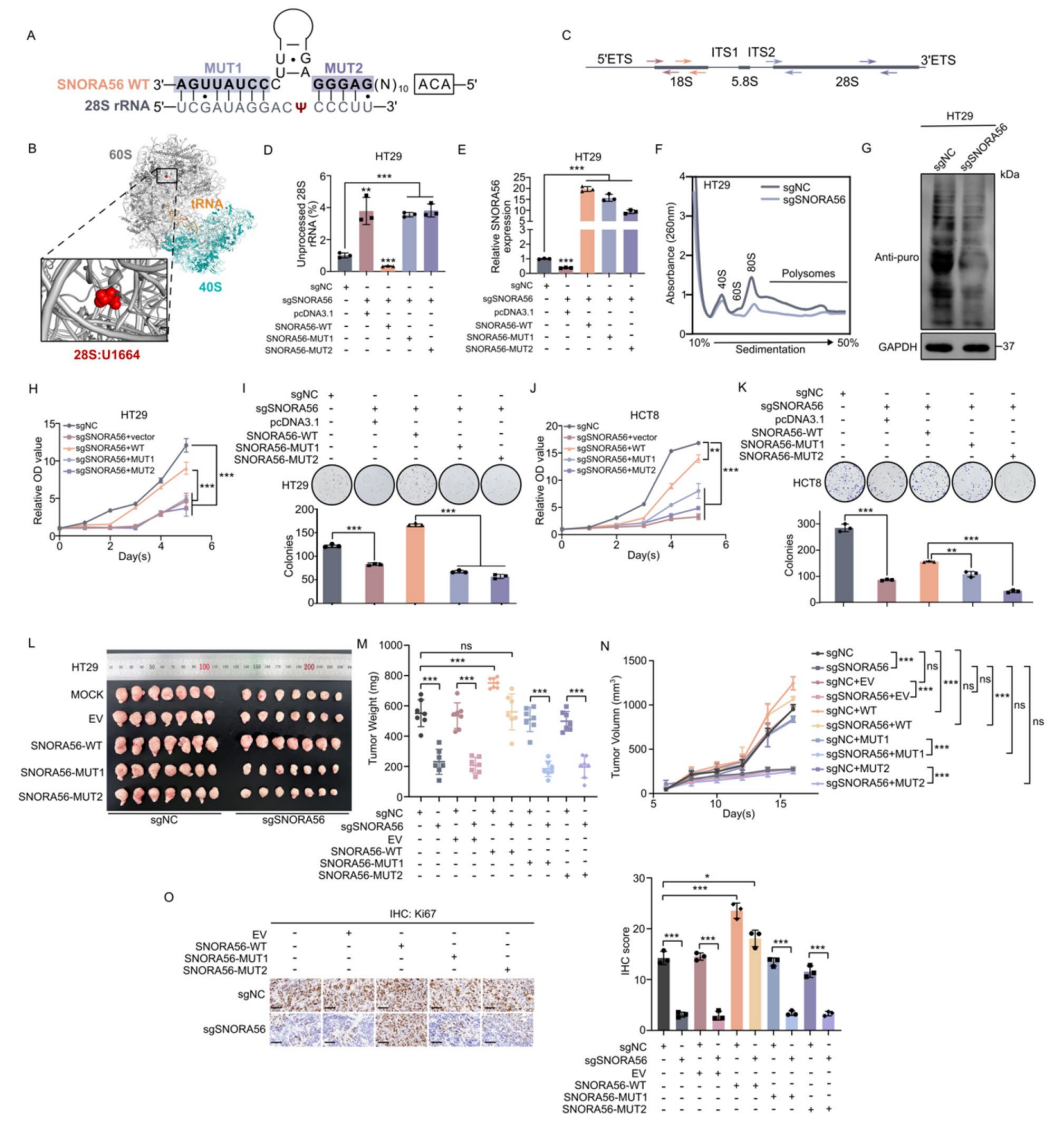
SNORA56 promotes 28 S rRNA maturation and translation in CRC cells. **(A)** Schematic representation of the interaction between SNORA56 and 28 S rRNA, and the mutated regions. **(B)** PyMOL analysis of the ribosomal structure and U1664, the 28 S rRNA site that is pseudouridylated by SNORA56. **(C)** A schematic diagram of the primers used to detect total and precursors of 18 and 28 S rRNA. Paired primers were displayed in the same color. **(D-E)** RT-qPCR analysis was used to reveal the relative levels of unprocessed 28 S rRNA and SNORA56 in HT29 sgNC and sgSNORA56 cells transfected with SNORA56-WT, MUT1, or MUT2. **(F)** Polysome profiling of HT29 cells with stable SNORA56 knockdown vs. the control. **(G)** OP-puro analysis of HCT29 cells with stable SNORA56 knockdown vs. the control. **(H–K)** CCK8 and colony formation assays of the treated HT29 and HCT8 cells. **(L)** Tumors from nude mice subcutaneous xenografts bearing HT29 sgNC, sgSNORA56, together with empty vector (EV), SNORA56-WT, MUT1 and MUT2, respectively. **(M–N)** Tumor weights and growth curves. **(O)** IHC analyses of Ki67 in the xenograft tumors. Scare bar: 100 μm

Because snoRNAs canonically function in ribosome modification, we first examined the effect of SNORA56 on rRNA maturation. According to the snoRNA Orthological Gene Database (snOPY), SNORA56 guides 28 S rRNA U1664 pseudouridylation via a base-pairing interaction between its flanking regions and rRNA (Fig. 3A). Structure analysis revealed that although the U1664 site is located in the large ribosomal subunit, it is far from the ribosome’s functional domain (Fig. 3B), implying that SNORA56 might alter ribosomal conformation. To investigate SNORA56-mediated pseudouridylation, we mutated two interacted flanking regions of SNORA56 to their complementary matched sequences respectively to disrupt the association between SNORA56 and 28 S rRNA as previously described (Fig. 3A, MUT1 and MUT2) [6, 30]. We then examined the expression levels of SNORA56 after transfection with its mutants or non-mutated control (WT) (Fig. 3E). Also, qPCR analysis using specific primers against unprocessed or total rRNAs revealed that SNORA56 silencing markedly increased the levels of immature 28 S rRNA (Fig. 3C–D, S4B), whereas SNORA56 overexpression reduced their level (Figure S4A). However, SNORA56 levels did not have observable effects on 18 S rRNA maturation (Figure S4C–E), which is consistent with the interaction between SNORA56 and 28 S rRNA not 18 S rRNA. Furthermore, polysome profiling and OP-Puro analysis of ribosomal activity showed that SNORA56 markedly enhances the translation capacity of the ribosome in CRC cells (Fig. 3F–G, S4F–G).

Next, to determine whether the tumorigenic role of SNORA56 is mediated by its interaction with 28 S rRNA, we overexpressed SNORA56-WT, MUT1 or MUT2, in SNORA56-depleted HCT8 and HT29 cells respectively (Fig. 3E, S4H–J). This analysis revealed that overexpressing SNORA56-WT, and not the mutants, restored the levels of 28 S rRNA (Fig. 3D, S4B), implying that interaction between SNORA56 and 28 S rRNA is essential for 28 S rRNA maturation. Notably, SNORA56 mutants failed to rescue SNORA56 silencing-induced proliferation both in vitro and in vivo (Fig. 3H–O), suggesting that SNORA56 plays a tumorigenesis role relying on the pseudouridylation of 28 S rRNA at U1664 site.

### SNORA56 upregulates GCLC protein expression by activating its translation

**Fig. 4:**
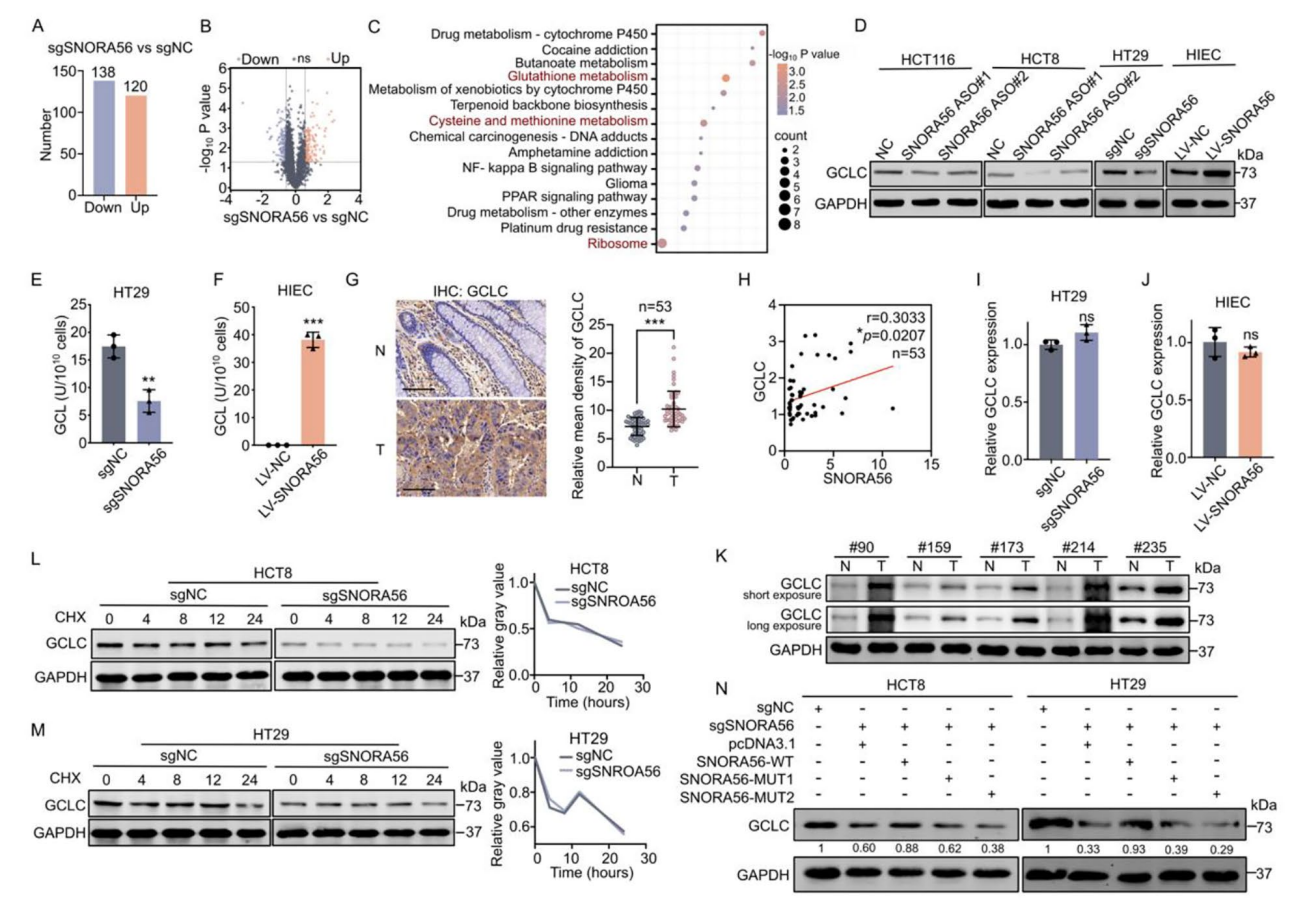
SNORA56 upregulates GCLC protein by activating its translation. **(A)** Differential protein expression in the sgSNORA56 vs. the sgNC group. **(B)** Volcano plot of differentially expressed proteins. **(C)** KEGG pathway enrichment analysis of significantly downregulated proteins. **(D)** GCLC protein levels in HCT116, HCT8, HT29 and HIEC cells were determined using western blotting after indicated transfections. **(E–F)** GCL enzyme activity in HT29 and HIEC cells with indicated transfection. **(G)** IHC analysis of GCLC levels in CRC tissue microarray. Mean GCLC density was determined using Image Pro Plus. Scale bar: 100 μm. **(H)** Analysis of the correlation between the SNORA56 and GCLC protein levels using CRC tissue microarray. **(I–J)** RT-qPCR analysis of relative GCLC mRNA levels in HT29 and HIEC cells upon indicated transfections. **(K)** Western blot analysis of GCLC protein levels in paired CRC tissues. **(L–M)** Western blot analysis of GCLC protein stability in SNORA56-silenced HCT8 and HT29 cells vs. control cells after CHX treatment for indicated durations. Relative band intensities were measured on ImageJ. **(N)** Western blot analysis of GCLC protein levels in HT29 and HCT8 cells after indicated transfections. GCLC protein levels were normalized to GAPDH.

To identify potential downstream targets of SNORA56 in CRC, we carried out a proteomic analysis on sgNC and sgSNORA56 cells (Fig. 2C–D). This analysis uncovered 258 differentially expressed proteins, of which 120 were upregulated and 138 were downregulated (Fig. 4A–B). Clusters of Orthologous Groups/Eukaryotic Orthologous Groups (COG/KOG) functional classification showed that 10 of the downregulated proteins are involved in translation, ribosomal structure, and biogenesis (Figure S5A). Moreover, KEGG functional enrichment analysis revealed that the downregulated proteins were enriched for the ribosome (Fig. 4C), further indicating that SNORA56 is involved in ribosome activation. Notably, the downregulated proteins were enriched for metabolic processes associated with ferroptosis, such as glutathione and cysteine and methionine metabolism (Fig. 4C), suggesting that SNORA56 might influence ferroptosis by regulating metabolism.

To test this possibility, we focused on the protein GCLC, an indispensable rate-limiting enzyme in the biosynthesis of glutathione. GCLC is downregulated upon SNORA56 silencing and it may inhibit ferroptosis by decreasing cellular peroxide levels. Consistently, transient or stable SNORA56 silencing in CRC cells significantly reduced GCLC protein levels (Fig. 4D, S5B). Furthermore, we found that SNORA56 deficiency significantly decreased the activity of glutamate cysteine ligase (Fig. 4E–F, S5C), whereas SNORA56 overexpression markedly upregulated GCLC protein and enzymatic levels (Fig. 4D, F). Moreover, IHC analysis of CRC tissue microarray revealed GCLC upregulation in paired CRC and adjacent non-tumor tissues (Fig. 4G). Further analysis indicated a significant positive correlation between GCLC protein level and SNORA56 staining intensity (Fig. 4H). Next, validation analysis of the relationship using other paired CRC tissues revealed that as with SNORA56, GCLC protein levels were markedly upregulated in tumors (Fig. 4K). However, GCLC mRNA levels were unaffected by SNORA56 (Fig. 4I–J, S5D–E), implying that SNORA56 regulates GCLC posttranscriptionally or translationally. To test this, GCLC protein stability was assessed upon treatment with cycloheximide (CHX) in HT29 and HCT8 cells to block protein synthesis and evaluate protein degradation. This analysis revealed no clear differences in GCLC protein half-life in conditions of SNORA56 deficiency or overexpression (Fig. 4L–M, S5F), suggesting that SNORA56 regulates GCLC translation and not degradation. To test this possibility, we transfected stable SNORA56-silenced HCT8 and HT29 cells with SNORA56-WT or with 28 S rRNA binding-impaired SNORA56 mutants. Importantly, the SNORA56 mutants failed to rescue GCLC protein expression (Fig. 4N), indicating that the SNORA56-induced maturation of 28 S rRNA was required for the high GCLC protein levels in CRC.

### SNORA56 inhibits ferroptosis and promotes proliferation by upregulating GCLC protein in CRC

**Fig. 5:**
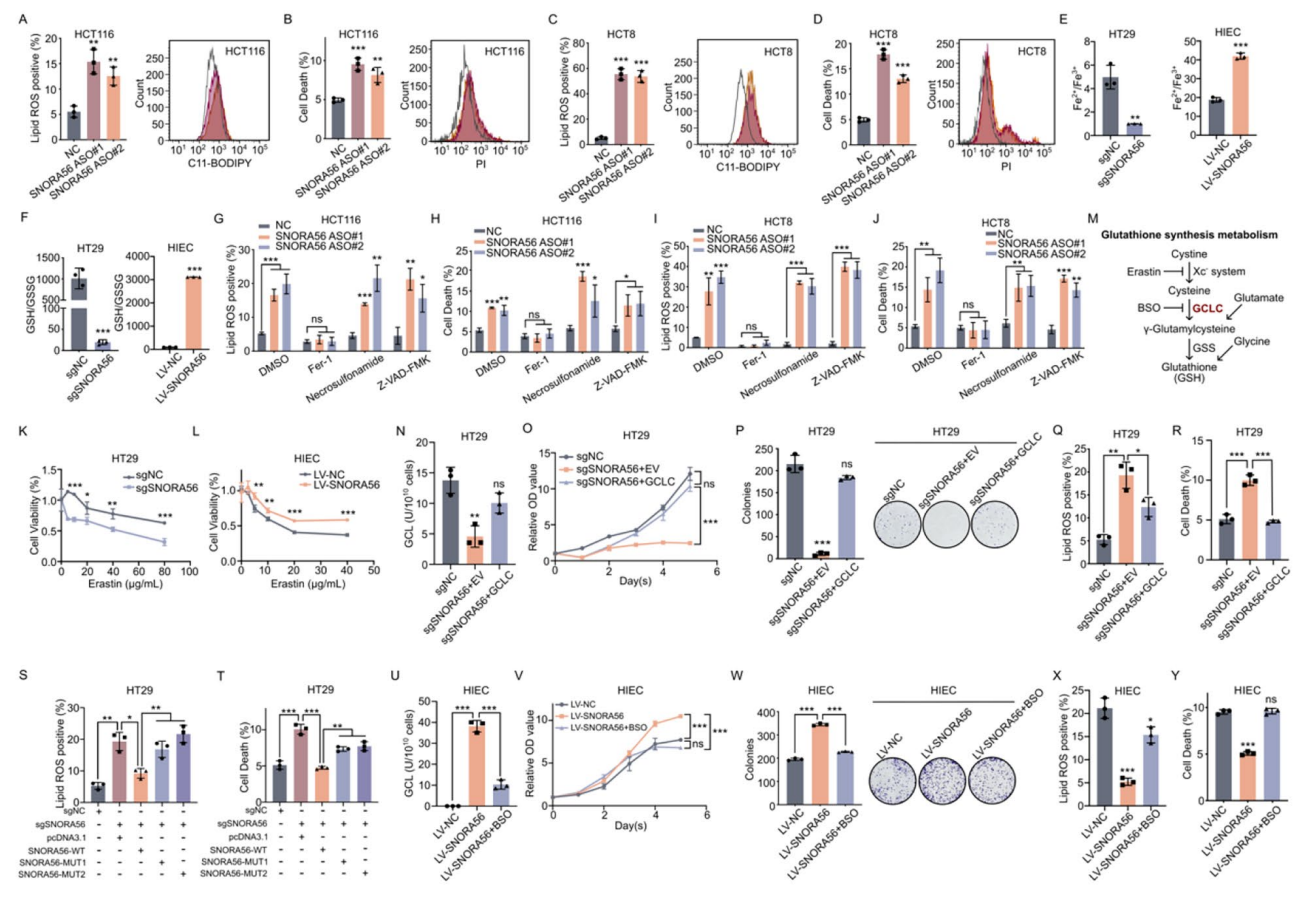
SNORA56 causes GCLC-mediated CRC ferroptosis resistance and proliferation. **(A, C, S, X, Q)** HCT116, HCT8, HT29, and HIEC cells were transfected as indicated, stained with the BODIPY C11 probe, and subjected to flow cytometry for lipid ROS detection. **(B, D, T, Y, R)** HCT116, HCT8, HT29, and HIEC cells were subjected to indicated transfections, followed by propidium iodide staining and cell death analysis using flow cytometry. **(E)** The relative Fe^2+^/Fe^3+^ ratios in HT29 and HIEC cells after indicated transfections. **(F)** The relative GSH/GSSG ratios in HT29 and HIEC cells after indicated transfections. **(G-J)** Lipid ROS levels and cell death in HCT116 and HCT8 cells transfected with SNORA56 ASOs and control ASO, followed by treatment with DMSO, Fer-1, necrosulfonamide, and Z-VAD-FMK, were measured using flow cytometry. **(K-L)** The cell viability of HT29 and HIEC cells after indicated transfections and treatment with various erastin concentrations for 24 h, was determined using the CCK8 assay. **(M)** A schematic representation of GSH synthesis. **(N, U)** GCL enzyme activity in HT29 and HIEC cells after indicated treatment. **(O-P, V-W)** CCK8 and colony formation assays were used to assess the proliferation of treated HT29 and HIEC cells

Because GCLC is thought to function in peroxide clearance, we investigated if SNORA56 regulates ferroptosis in CRC. Lipid reactive oxygen species (ROS) and cell death were assessed using flow cytometric analysis of HCT116, HCT8 and HT29 cells after transient or stable SNORA56 silencing. Notably, our data showed that SNORA56 deficiency markedly caused lipid ROS accumulation accompanied by increased cell death (Fig. 5A–D, S6A–D), suggesting that SNORA56 is involved in ferroptosis inhibition in CRC cells. Because labile iron contributes to ferroptosis by directly oxidizing lipids via Fenton reaction and acting as a cofactor for lipid oxidizing enzymes [31], we assessed the Fe^2+^/Fe^3+^ ratio to validate the role of SNORA56 in ferroptosis regulation. This analysis revealed a marked decrease in the Fe^2+^/Fe^3+^ ratio in the SNORA56 knockdown group (Fig. 5E, S6E), which is consistent with peroxidation in the cellular microenvironment. Similarly, the GSH/GSSG ratio was reduced by SNORA56 silencing (Fig. 5F, S6F), indicating that SNORA56 depletion triggers ferroptosis in CRC cells. However, SNORA56 overexpression in HIEC cells increased both ratios and resistance to erastin, a canonical ferroptosis inducer that blocks the system Xc^-^ antiporter (Fig. 5E-F, L, S6G–H). Notably, only the ferroptosis inhibitor, ferrostatin-1 (Fer-1) reversed lipid ROS elevation and cell death following SNORA56 deficiency, and both the necroptosis inhibitor, necrosulfonamide, and the apoptosis inhibitor, Z-VAD-FMK, could not prevent CRC cell death (Fig. 5G–J). Moreover, analysis of the impact of SNORA56 on ferroptosis sensitivity in CRC revealed that SNORA56 downregulation markedly sensitized CRC cells to ferroptosis in a dose-dependent manner, whereas SNORA56 overexpression caused ferroptosis resistance (Fig. 5K-L, S6L). These observations highlight targeting both SNROA56 and ferroptosis inducers as a promising strategy for CRC treatment. Additionally, cell viability assays revealed that SNORA56 knockdown suppressed cell growth, which could be almost entirely restrained after treatment with Fer-1 (Figure S6I–K), implying that SNORA56 facilitates cell proliferation, at least partly, through ferroptosis resistance. These data illustrate that SNORA56 mediates ferroptosis resistance and highlight SNORA56 as a critical target for regulating ferroptosis sensitivity in CRC cells.

Since GCLC is a key rate-limiting enzyme in GSH synthesis (Fig. 5M), we next evaluated whether SNORA56-mediated ferroptosis inhibition depends on GCLC protein expression. To determine the hypothesis, we generated a GCLC overexpression system after silencing SNORA56 and verified transfection efficiency using RT-qPCR and western blotting (Figure S6M–P). As expected, GCLC overexpression rescued GCL enzyme activity in HT29 cells (Fig. 5N, S6Q), and remarkably, CRC cell proliferation was reversed upon GCLC overexpression (Fig. 5O–P, S6R–S). Moreover, lipid ROS, cell death, and ferroptosis sensitivity were partially reversed under GCLC retrieved (Fig. 5Q-R, S6T–U, X-Y), indicating that SNORA56 contributes to ferroptosis resistance in CRC mediated by GCLC. Considering SNORA56 facilitates GCLC translation via the 28 S rRNA pseudouridylation, we transfected with SNORA56-WT, MUT1 or MUT2 respectively in HCT8 and HT29 sgSNORA56 cells and examined the ferroptosis. As expected, SNORA56 mutants with depleted 28 S rRNA binding capability failed to recover the ferroptosis resistance (Fig. 5S-T, S6V-W). Next, we examined the pharmacological effect of DL-Buthionine-Sulfoximine (BSO), which inhibits glutathione synthesis and induces ferroptosis, in SNORA56-overexpressing HIEC cells. This analysis revealed that BSO markedly suppressed SNORA56-induced elevation of GCL enzyme activity (Fig. 5U), as well as SNORA56-driven proliferation and ferroptosis inhibition in HIEC cells (Fig. 5V–Y), indicating that SNORA56 promotes CRC tumorigenesis by enhancing GSH protein synthesis.

### SNORA56 is a promising CRC diagnostic biomarker and therapeutic target

**Fig. 6:**
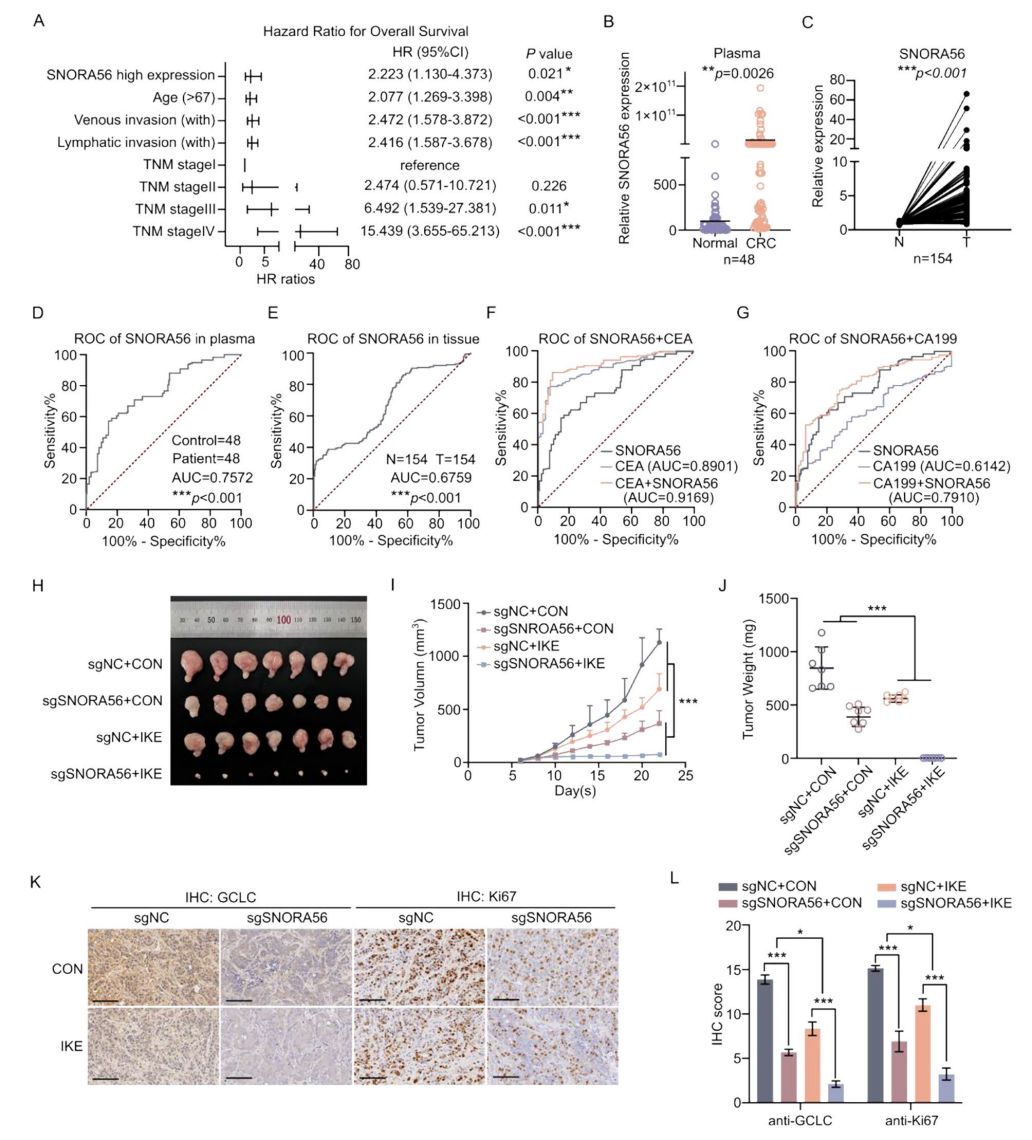
SNORA56 is a promising CRC diagnostic biomarker and therapeutic target. **(A)** Forest plot of the overall survival hazard ratios using TCGA_COAD data. **(B)** Relative levels of plasma SNORA56. **(C)** Relative SNORA56 expression in 154 paired CRC tissues was revealed by RT-qPCR. **(D–E)** ROC curve analysis of SNORA56 in CRC plasma and tissues. **(F–G)** ROC curve analysis of the CRC diagnostic potential of CEA or CA199 when combined with SNORA56. **(H)** Tumors from a nude mouse xenograft model that was subcutaneously injected with HT29 sgNC (right) and sgSNORA56 (left) cells and then treated with IKE or solvent respectively. **(I)** Plots of tumor growth measurements taken on indicated days. **(J)** Tumor weights. **(K)** IHC analysis of GCLC and Ki67 in xenograft tumors after treatment with IKE or the solvent. Scale bar: 100 μm. **(L)** GCLC and Ki67 IHC scores in indicated xenografts

To further investigate the correlation between SNORA56 expression and the clinical characteristics of CRC patients, we obtained SNORA56 expression data from the TCGA_COAD dataset (n = 394) from SNORic, along with clinical data from the UCSC Xena website and analyzed it using chi-square tests or Fisher’s exact tests to illustrate the potential relationship between SNORA56 level and clinical index. These analyses revealed a significant correlation between SNORA56 expression and histological type (*P* = 0.015), venous invasion (*P* = 0.040), and distant metastasis (*P* = 0.002) (Table S1–2). Importantly, Cox proportional hazards regression analysis identified high SNORA56 expression as an independent risk factor for CRC patients’ overall survival (HR = 2.223, *P* = 0.021, Fig. 6A). Because snoRNAs are stable in the plasma, we assessed SNORA56 levels in the plasma of CRC patients (age: 66.61 ± 11.55, n = 48) vs. healthy subjects without underlying gastrointestinal disease (age: 57.72 ± 8.67, n = 48). This analysis revealed higher SNORA56 levels in CRC plasma (Fig. 6B). We then collected 154 paired CRC tissues to evaluate the diagnosis efficacy of SNORA56. Consistent with this, SNORA56 levels were upregulated in CRC compared to the corresponding adjacent non-tumor tissues (Fig. 6C). To evaluate the diagnostic efficacy of SNORA56, analyses of the ROC curve were performed in CRC tissues and plasma, and the area under the curve (AUC) were 0.6759 and 0.7572, respectively (Fig. 6D–E), indicating that plasma SNORA56 has significant potential for CRC diagnosis. Notably, combining CEA or CA199, the canonical plasma-based CRC biomarkers, with SNORA56 markedly increased their AUC values (Fig. 6E–F), implying improved diagnostic value.

Finally, we examined the therapeutic potential of targeting SNORA56 in CRC. Because SNORA56 is involved in ferroptosis resistance, we first generated a xenograft model of CRC by subcutaneously injecting HT29 sgNC and sgSNORA56 cells into nude mice. Next, we intraperitoneally injected the mice with imidazole ketone erastin (IKE), an erastin substitute that is stable in vivo. Notably, knocking down SNORA56 markedly enhanced the sensitivity of the HT29 cells to IKE, characterized by significant suppression in tumor volumes and weights (Fig. 6H–J), highlighting the anti-CRC therapeutic potential of targeting SNORA56 while inducing ferroptosis. Moreover, GCLC and Ki67 staining was significantly weaker in the HT29 sgSNOR56 xenograft tumors than in the control, and this effect was enhanced by IKE (Fig. 6K–L), implying that SNORA56 exerts its effects on ferroptosis resistance and proliferation inhibition via GCLC. Together, these data highlight the diagnostic and therapeutic potential of SNORA56 in CRC.

**Fig. 7:**
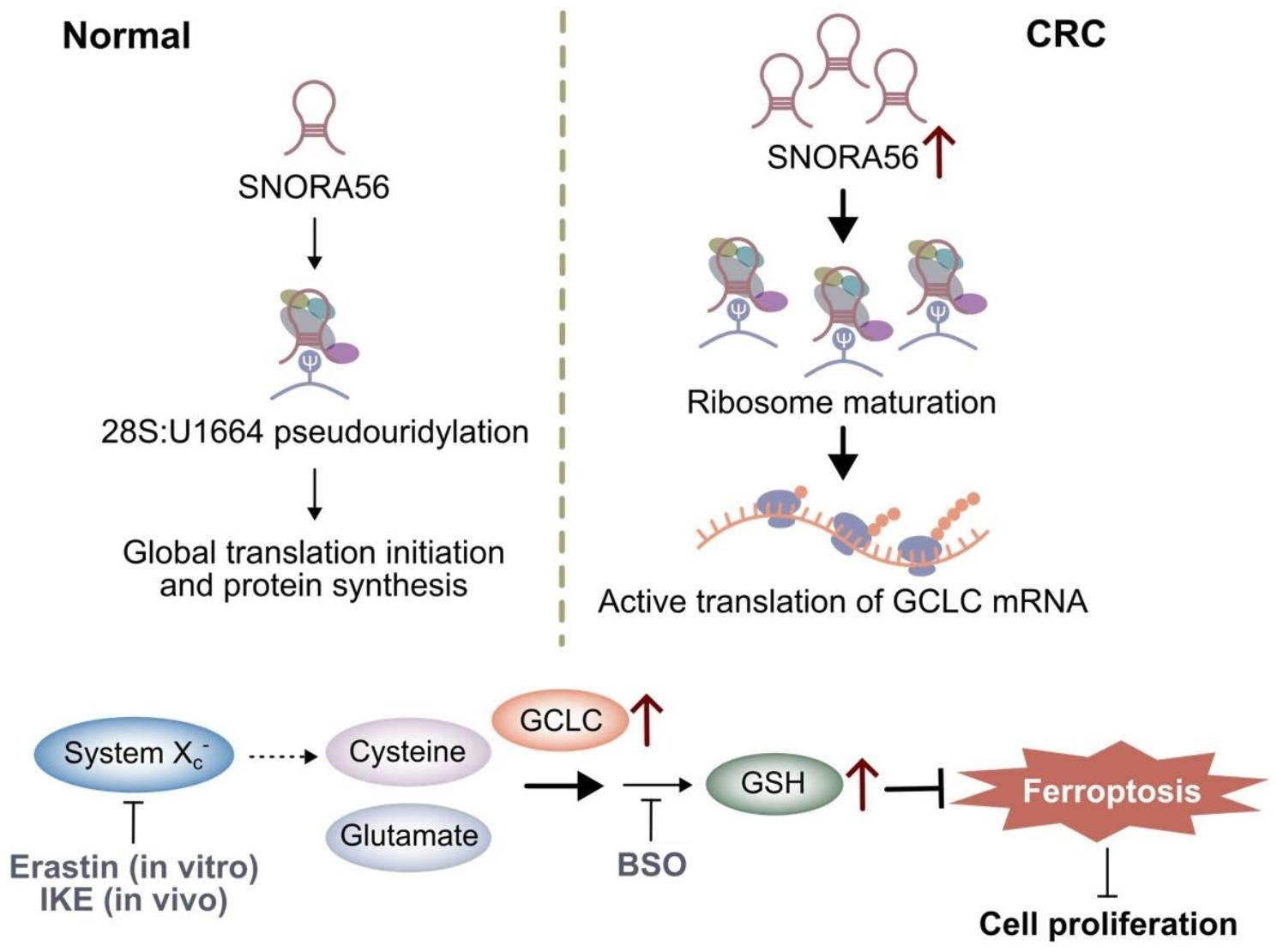
The proposed model of how SNORA56 regulates GCLC translation, thereby inhibiting ferroptosis and promoting proliferation. SNORA56, which was significantly upregulated in CRC, pseudouridylates 28 S rRNA at site U1664, thereby promoting ribosome maturation. Consequently, SNORA56 triggers GCLC translation, which drives ferroptosis resistance and proliferation in CRC.

## Discussion

Previous studies have reported that snoRNAs regulate rRNA modification, RNA splicing, and translation to affect cell fate, mainly through RNA 2’-O-methylation and pseudouridylation. The function of snoRNAs is dysregulated in various cancers and is a promising biomarker for cancer diagnosis and prognosis. Here, we for the first time, show that SNORA56 plays critical roles in CRC pathogenesis. Our data show marked SNORA56 upregulation in CRC was correlated with poor prognosis, which is consistent with its host gene DKC1^27^. Notably, SNORA56 stimulated ferroptosis resistance and promoted CRC proliferation in vivo and in vitro independently of DKC1.

Pseudouridylation, the most common posttranscriptional modification in non-coding RNAs, is mainly executed by box H/ACA snoRNAs that have two hairpins containing an internal pseudouridylation loop. SNORA56 is reported to mediate 28 S rRNA pseudouridylation at the U1664 site via base-pair interactions [32]. Interestingly, a recent report proposed that H/ACA pseudouridylation loops can consecutively synthesize two pseudouridines [33], which offers versatility in function of SNORAs apart from stabilizing RNA structure canonically. Our results revealed that SNORA56 mutants with complementary pairing interaction regions that lack rRNA binding activity, are associated with increased levels of unprocessed 28 S rRNA and reduced carcinogenesis, indicating that the oncogenic roles of SNORA56 in CRC are partly attributable to its effect on ribosome maturation. As expected, SNROA56 silencing, which impaired pseudouridylation at site 28 S-U1664, globally suppressed translation. However, our structural analyses showed that site 28 S-U1664 is located far from the ribosome’s decoding region, indicating that SNORA56 might not directly affect translation and that it was more likely to regulate translation by altering ribosome conformation and promoting ribosome structural stability.

Because the formation of pseudouridine affects ribosomal biogenesis and translation [34], we performed proteomics analysis using CRC cells and found that several ribosomal proteins were markedly decreased upon SNORA56 silencing. Notably, we found that ribosomal proteins, especially 60 S-associated factors like RPL4, RPL28, RPL35A, and RPL17, were markedly downregulated during SNORA56 deficiency, further highlighting the importance of SNORA56 in ribosome biogenesis in CRC. Analysis of ribosome function using polysome profiling and the OP-Puro assay showed that upon SNORA56 depletion, the 60 S subunit was inactivated, which was accompanied by reduced nascent peptide levels. Together, these findings indicated that SNORA56-mediated 28 S-U1664 pseudouridylation is required for ribosome assembly and global translation in CRC cells.

Next, we assessed the effect of SNORA56 on the translation of GCLC, one of its main downstream candidates. This analysis confirmed that SNORA56 elevated GCLC protein levels but did not affect its transcription. Moreover, SNORA56 did not significantly influence GCLC protein stability, indicating that its effect on GCLC is not dependent on protein degradation. Specifically, GCLC protein levels fully relied on the binding of the SNORA56 pseudouridylation loop to 28 S rRNA, implying that SNORA56 may affect GCLC translation by regulating U1664 pseudouridylation. Hundreds of pseudouridylation mRNA sites have been identified and shown to respond to environmental alterations [35]. Therefore, it is possible that in some circumstances, SNORA56 promotes ferroptosis resistance and proliferation in CRC by directly inducing the pseudouridylation of specific mRNAs. Moreover, we predicted the interaction between the SNORA56 pseudouridylation loop and the GCLC mRNA sequence using the online tool, IntaRNA (http://rna.informatik.uni-freiburg.de/IntaRNA/Input.jsp). In the future, we will explore whether SNORA56 regulates GCLC translation by controlling its mRNA pseudouridylation.

GCL, an indispensable rate-limiting enzyme in GSH biosynthesis composed of catalyzed subunit GCLC and modifier subunit GCLM, catalyzes the ligation of glutamate and cysteine in the first step [36]. In this study, we found that SNORA56 mediates CRC ferroptosis resistance and proliferation at least in part, by regulating GCLC protein expression. Intriguingly, GCLM protein also decreased after SNORA56 silencing according to our proteomics analysis, indicating that SNORA56 may play a dual promoting role both in translation and in the affinity to glutamate of GCLC. Ferroptosis, a form of programmed cell death characterized by lipid peroxidation, is triggered when the antioxidant status is compromised [31]. Mounting evidence indicates that GCLC suppresses ferroptosis through the reductive effects of GSH [14, 16, 37]. Notably, ferroptosis involves multiple metabolic processes and also influences response to cancer chemotherapy, radiotherapy, and immunotherapy [18, 38, 39]. Thus, targeting GSH metabolism to induce ferroptosis is a promising potential strategy for CRC therapy. Although metabolic reprogramming is a hallmark of cancer, interventions that target cancer metabolism have not been highly successful in clinical trials because of its flexibility and heterogeneity. A recent study demonstrated that deubiquitination can maintain protein homeostasis, which allows cancer cells to survive when GSH is depletion [40]. Therefore, to identify potential therapeutic strategies, it is necessary to determine the mechanisms that underlie cancer metabolic adaptation. Numerous studies have indicated that the transcription of GCLC is controlled by nuclear factor erythroid 2-related factor 2 signaling [37, 41, 42], and that it correlates with prognosis in various cancers [43, 44]. Here, our findings propose a novel regulatory mechanism through which SNORA56 enhances GCLC translation, thereby inhibiting ferroptosis and promoting CRC proliferation. Thus, combining SNORA56 targeting and ferroptosis induction may be a potential CRC treatment strategy. However, the precise reasons for the regulation of SNORA56 expression, and the efficacy of such combined therapy, need further investigation.

## Conclusions

Taken together, our data show that SNORA56 is a key factor in CRC pathogenesis (Fig. 7). We found that SNORA56 was markedly elevated in CRC tissue and plasma and that it is a promising biomarker for CRC diagnosis and prognosis. Our study validated that SNORA56 stimulates CRC ferroptosis resistance and promotes CRC proliferation in vitro and in vivo. Importantly, we found that SNORA56 pseudouridylated 28 S rRNA at the U1664 site, which activated GCLC translation. GCLC, a rate-limiting enzyme in GSH synthesis, inhibited ferroptosis, thereby further enhancing CRC proliferation. These findings provide novel insights into SNORA56 regulation and highlight it as a potential target for combined CRC therapy.

### Electronic supplementary material

Below is the link to the electronic supplementary material.

Supplementary Material 1: Tables 1–2.pdf.

Supplementary Material 2: Reagent and resource.pdf.

Supplementary Material 3: Figs. 1–6.pdf.

## Data Availability

The datasets used or analyzed during the current study are available from the corresponding author on reasonable request.

## List of Abbreviations

CRC: Colorectal cancer
GCLC: Glutamate cysteine ligase catalytic subunit
snoRNAs: small nucleolar RNAs
GSH: Reduced glutathione
GSSG: Oxidized glutathione
DKC1: Dyskerin 1
ASOs: Antisense oligonucleotides
TCGA: The Cancer Genome Atlas
RT-qPCR: Real-time quantitative PCR
FISH: Fluorescence in situ hybridization
IHC: Immunohistochemistry
H&E: Hematoxylin & eosin
OP-Puro: O-propargyl-puromycin
CCK8: Cell counting kit-8
ROC: Receiver operating characteristic
AUC: Area under the curve
CEA: Carcinoembryonic antigen
CA199: Carbohydrate antigen199
CHX: Cycloheximide
ROS: Reactive oxygen species
Fer-1: Ferrostatin-1
IKE: Imidazole ketone erastin
BSO: DL-Buthionine-Sulfoximine

## References

[1] Sung H (2021). Global Cancer statistics 2020: GLOBOCAN estimates of incidence and Mortality Worldwide for 36 cancers in 185 countries. CA A Cancer J Clin.

[2] Patel SG, Karlitz JJ, Yen T, Lieu CH, Boland CR (2022). The rising tide of early-onset Colorectal cancer: a comprehensive review of epidemiology, clinical features, biology, risk factors, prevention, and early detection. Lancet Gastroenterol Hepatol.

[3] Dekker E, Tanis PJ, Vleugels JLA, Kasi PM, Wallace MB (2019). Colorectal cancer. The Lancet.

[4] Williams GT, Farzaneh F (2012). Are snoRNAs and snoRNA host genes new players in cancer?. Nat Rev Cancer.

[5] Wang K (2023). SNORD88C guided 2′-O-methylation of 28S rRNA regulates SCD1 translation to inhibit autophagy and promote growth and Metastasis in non-small cell Lung cancer. Cell Death Differ.

[6] Zhu J (2023). SNORA14A inhibits hepatoblastoma cell proliferation by regulating SDHB-mediated succinate metabolism. Cell Death Discov.

[7] Liu Z (2021). SNORA23 inhibits HCC tumorigenesis by impairing the 2’-O-ribose methylation level of 28S rRNA. Cancer Biol Med.

[8] Zhuo Y (2022). Targeting SNORA38B attenuates tumorigenesis and sensitizes immune checkpoint blockade in non-small cell Lung cancer by remodeling the Tumor microenvironment via regulation of GAB2/AKT/mTOR signaling pathway. J Immunother Cancer.

[9] Wu H (2020). Long noncoding RNA ZFAS1 promoting small nucleolar RNA-mediated 2′-O-methylation via NOP58 recruitment in Colorectal cancer. Mol Cancer.

[10] Liao J (2010). Small nucleolar RNA signatures as biomarkers for non-small-cell Lung cancer. Mol Cancer.

[11] Sedlak JC, Yilmaz ÖH, Roper J (2023). Metabolism and Colorectal Cancer. Annu Rev Pathol Mech Dis.

[12] Balihodzic A (2022). Non-coding RNAs and ferroptosis: potential implications for cancer therapy. Cell Death Differ.

[13] Nguyen A (2016). PKLR promotes Colorectal cancer liver colonization through induction of glutathione synthesis. J Clin Invest.

[14] Kang YP (2021). Non-canonical glutamate-cysteine ligase activity protects against Ferroptosis. Cell Metabol.

[15] Laoukili J (2022). Peritoneal metastases from Colorectal cancer belong to Consensus Molecular Subtype 4 and are sensitised to oxaliplatin by inhibiting reducing capacity. Br J Cancer.

[16] Yang Z (2023). ACTL6A protects gastric cancer cells against ferroptosis through induction of glutathione synthesis. Nat Commun.

[17] Fernández-Ginés R (2022). An inhibitor of interaction between the transcription factor NRF2 and the E3 ubiquitin ligase adapter β-TrCP delivers anti-inflammatory responses in mouse liver. Redox Biol.

[18] Zhang H (2020). CAF secreted miR-522 suppresses ferroptosis and promotes acquired chemo-resistance in gastric cancer. Mol Cancer.

[19] Zhang Y, Luo M, Cui X, O’Connell D, Yang Y (2022). Long noncoding RNA NEAT1 promotes ferroptosis by modulating the miR-362-3p/MIOX axis as a ceRNA. Cell Death Differ.

[20] Tang Y, Li C, Zhang Y-J, Wu Z-H (2021). Ferroptosis-related long non-coding RNA signature predicts the prognosis of Head and neck squamous cell carcinoma. Int J Biol Sci.

[21] Wu Z (2021). Identification and Validation of Ferroptosis-Related LncRNA Signatures as a novel prognostic model for Colon Cancer. Front Immunol.

[22] Tang R (2022). Ferroptosis-related lncRNA pairs to predict the clinical outcome and molecular characteristics of pancreatic ductal adenocarcinoma. Brief Bioinform.

[23] Concordet J-P, Haeussler M (2018). CRISPOR: intuitive guide selection for CRISPR/Cas9 genome editing experiments and screens. Nucleic Acids Res.

[24] Liu L et al. The N6-methyladenosine modification enhances ferroptosis resistance through inhibiting *SLC7A11* mRNA deadenylation in hepatoblastoma. *Clinical & Translational Med* 12, (2022). 10.1002/ctm2.778 PMC9076012 35522946

[25] Zhang Z et al. SNORA71A Promotes Colorectal Cancer Cell Proliferation, Migration, and Invasion. *Biomed Res Int* 2020, 8284576 (2020). 10.1155/2020/8284576 PMC7559222 33083486

[26] Skrzypczak M (2010). Modeling oncogenic signaling in colon tumors by multidirectional analyses of microarray data directed for maximization of analytical reliability. PLoS ONE.

[27] Kan G (2021). Dual inhibition of DKC1 and MEK1/2 synergistically restrains the growth of Colorectal Cancer cells. Adv Sci (Weinh).

[28] Gong J (2017). A pan-cancer analysis of the expression and clinical relevance of small nucleolar RNAs in Human Cancer. Cell Rep.

[29] Bian Z (2023). SNORD11B-mediated 2’-O-methylation of primary let-7a in colorectal carcinogenesis. Oncogene.

[30] Jinn S (2015). snoRNA U17 regulates Cellular cholesterol trafficking. Cell Metabol.

[31] Jiang X, Stockwell BR, Conrad M (2021). Ferroptosis: mechanisms, biology and role in Disease. Nat Rev Mol Cell Biol.

[32] Kiss AM, Jády BE, Bertrand E, Kiss T (2004). Human box H/ACA pseudouridylation guide RNA machinery. Mol Cell Biol.

[33] Jády BE, Ketele A, Moulis D, Kiss T, Guide (2022). RNA acrobatics: positioning consecutive uridines for pseudouridylation by H/ACA pseudouridylation loops with dual guide capacity. Genes Dev.

[34] Penzo M, Montanaro L (2018). Turning uridines around: role of rRNA pseudouridylation in Ribosome Biogenesis and ribosomal function. Biomolecules.

[35] Schwartz S (2014). Transcriptome-wide mapping reveals widespread dynamic-regulated pseudouridylation of ncRNA and mRNA. Cell.

[36] Misra I, Griffith OW (1998). Expression and purification of human gamma-glutamylcysteine synthetase. Protein Expr Purif.

[37] Xu Y, Li Y, Li J, Chen W (2022). Ethyl carbamate triggers ferroptosis in liver through inhibiting GSH synthesis and suppressing Nrf2 activation. Redox Biol.

[38] Koppula P (2022). A targetable CoQ-FSP1 axis drives ferroptosis- and radiation-resistance in KEAP1 inactive Lung Cancers. Nat Commun.

[39] Zhao L (2022). Ferroptosis in cancer and cancer immunotherapy. Cancer Commun (Lond).

[40] Harris IS (2019). Deubiquitinases maintain protein homeostasis and survival of Cancer cells upon glutathione depletion. Cell Metabol.

[41] You G-R et al. MYH9 Facilitates Cell Invasion and Radioresistance in Head and Neck Cancer via Modulation of Cellular ROS Levels by Activating the MAPK-Nrf2-GCLC Pathway. *Cells* 11, (2022). 10.3390/cells11182855 PMC9497050 36139430

[42] Huseby N-E, Ravuri C, Moens U (2016). The proteasome inhibitor lactacystin enhances GSH synthesis capacity by increased expression of antioxidant components in an Nrf2-independent, but p38 MAPK-dependent manner in rat colorectal carcinoma cells. Free Radic Res.

[43] Wang D (2021). Identification of the prognostic value of ferroptosis-related gene signature in Breast cancer patients. BMC Cancer.

[44] Luo L, Zhang Z, Weng Y, Zeng J. Ferroptosis-Related Gene GCLC Is a Novel Prognostic Molecular and Correlates with Immune Infiltrates in Lung Adenocarcinoma. *Cells* 11, (2022). 10.3390/cells11213371 PMC9657570 36359768

