# Supplementary material for: SNORA56-mediated pseudouridylation of 28 S rRNA inhibits ferroptosis and promotes colorectal cancer proliferation by enhancing GCLC translation
Source: J Exp Clin Cancer Res. 2023 Dec 5;42:331. doi: 10.1186/s13046-023-02906-8 (PMC10696674; doi:10.1186/s13046-023-02906-8)
Supplement: Supplementary file 1 — Supplementary Material 1: Tables 1–2.pdf. [file 13046_2023_2906_MOESM1_ESM.pdf]

Table S1. Characteristics of TCGA_COAD patients for SNORA56 expression in tumor tissues.

Parameters	Samples, *n*	*SNORA56* expression RPKM (Interquartile range)	*P*-value
**Age (years)**			0.3167
＞67	216	44.88 (20.190-98.335)	
≤67	178	22.29 (14.458-102.52)	
Unavailable	2		
**Gender**			0.4417
Male	207	39.07 (17.540 - 95.450)	
Female	187	43.47 (22.310-99.740)	
Unavailable	2		
**Histology**			0.1309
Adenomas and Adenocarcinomas	333	44.84 (19.165-106.57)	
Cystic, Mucinous and Serous Neoplasms	58	30.99 (15.013-60.830)	
Epithelial Neoplasms	3	95.51 (56.525-117.52)	
Unavailable	2		
**Lymphatic invasion**			0.7571
YES	161	43.10 (20.145-115.72)	
NO	211	45.63 (19.220-91.080)	
Unavailable	24		
**Venous invasion**			0.0620
YES	85	48.94 (25.020-116.46)	
NO	256	38.57 (16.270-88.838)	
Unavailable	55		
**T stage**			0.3875
T1	9	58.23 (32.265-206.27)	
T2	62	39.75 (19.273-95.923)	
T3	273	42.16 (17.700-106.98)	
T4	49	39.07 (23.880-82.375)	
Unavailable	3		
**LN meta.**			0.1374
N0	228	41.83 (17.870-90.700)	
N1	90	33.97 (13.188-103.688)	
N2	76	57.32 (25.173-118.968)	
Unavailable	2		

**Distant meta.**			****0.0022**
M0	286	44.67 (19.018-103.81)	
M1	57	58.36 (24.445-120.62)	
MX	45	27.81 (8.8950-53.680)	
Unavailable	8		
**TNM stage**			0.3316
I	61	38.67 (18.440-93.035)	
II	154	42.16 (17.560-88.920)	
III	113	37.39 (15.740-104.24)	
IV	57	58.36 (24.225-120.62)	
Unavailable	11		

RPKM, Reads Per Kilobase per Million mapped reads.

Table S2. The correlation analysis of SNORA56 expression and the clinical characteristics in 394 TCGA_COAD patients.

Parameters	SNORA56 expression#		$\chi2$	*p*-value
	Low	High		
**Age (years)**				
＞67	150	66	0.616	0.432
≤67	117	61		
**Gender**				
Male	144	63	0.646	0.422
Female	123	64		
**Histology**				
Adenomas and Adenocarcinomas	218	115		
Cystic, Mucinous and Serous Neoplasms	48	10	8.404	***0.015**
Epithelial Neoplasms	1	2		
**Lymphatic invasion**				
YES	102	59	1.645	0.200
NO	147	64		
**Venous invasion**				
YES	51	34	4.201	***0.040**
NO	184	72		
**TNM stage**				
I / II	157	66	1.636	0.201
III/ IV	110	61		

#Cutoff threshold of SNORA56 level was the average value in all patients.
