# Supplementary material for: SNORA56-mediated pseudouridylation of 28 S rRNA inhibits ferroptosis and promotes colorectal cancer proliferation by enhancing GCLC translation
Source: J Exp Clin Cancer Res. 2023 Dec 5;42:331. doi: 10.1186/s13046-023-02906-8 (PMC10696674; doi:10.1186/s13046-023-02906-8)
Supplement: Supplementary file 2 — Supplementary Material 2: Reagent and resource.pdf. [file 13046_2023_2906_MOESM2_ESM.pdf]

1. **Oligonucleotides**

SNORA56 ASO#1 TTCTTCTGTCCAGCGTCAGT

SNORA56 ASO#2 ATCCCTTTCTAGTCTGGCTC

ASO#NC was supported by RIBOBIO corporation.

gRNA-SNORA56-Oligo-Forward:

5'CACCG TCCCACGAGCCAGACTAGAA3'

gRNA-SNORA56-Oligo-Reverse:

5'AAACTTCTAGTCTGGCTCGTGGGAC3'

SNORA56 FISH probe:

5'Dig-TCCCTCTAGAGTCCCACGAGCCAGACTAGAAAG-Dig3'

qPCR Primers:

18S-Forward: 5' CAGCCACCCGAGATTGAGCA 3'

18S-Reverse: 5' TAGTAGCGACGGGCGGTGTG 3'

U6-Forward: 5' GTGCTCGCTTCGGCAGCACATATAC 3'

U6-Reverse: 5' AAAAATATGGAACGCTCACGAATTTG 3'

SNORA1-Forward: 5' CTAGAGAATGGGCACTGTTGA 3'

SNORA1-Reverse: 5' TGTTACAGGTCTCATTGTCAC 3'

SNORA27-Forward: 5' TACCCCCTTTTCACTTTGCC 3'

SNORA27-Reverse: 5' CCTCTGACAGGATACAGACAA 3'

SNORD18B-Forward: 5' GATGAGATTCCACTTAATTGG 3'

SNORD18B-Reverse: 5' CAAGTCAGAATTTCCACA 3'

SNORA56-Forward: 5' CAGACAGTTATCCCTTTCTAG 3'

SNORA56-Reverse: 5' TGTTATCTGGCAAGTCTAAAG 3'

DKC1-Forward: 5' GCTAAGTTGGACACGTCTCAG 3'

DKC1-Reverse: 5' TGCAAGAGGTGTATAGTGTGTTG 3'

GCLC-Forward: 5' GGAGGAAACCAAGCGCCAT 3'

GCLC-Reverse: 5' CTTGACGGCGTGGTAGATGT 3'

unprocessed 18S-Forward: 5' CTCGCCGCGCTCTACCTTACCTACCTGG 3'

unprocessed 18S-Reverse: 5' GCGCCCGTCGGCATGTATTAGCTC 3'

total 18S-Forward: 5' GGCCCTGTAATTGGAATGAG 3'

total 18S-Reverse: 5' GCGGGACACTCAGCTAAGAGC 3'

unprocessed 28S-Forward: 5' CCCGTCCCCCTCCGAGACGCGACC 3'

unprocessed 28S-Reverse: 5' CGCTGGGCTCTTCCCTGTTCACTCG 3'

total 28S-Forward: 5' CCAAGTCCTTCTGATCGAGGCCC 3'

total 28S-Reverse: 5' CTTACGGTACTTGTTGACTATCGGTCTCG 3'

2. Plasmids

lentiCRISPR v2

psPAX2

pMD2.G

pLVX-AcGFP

pLVX-AcGFP-SNORA56

pLVX-AcGFP-SNORA56 MUT1

pLVX-AcGFP-SNORA56 MUT2

pCDNA3.1-3×FLAG

pCDNA3.1-3×FLAG-GCLC

pCDNA3.1(+)

pCDNA3.1-SNORA56 WT

pCDNA3.1-SNORA56 MUT1

pCDNA3.1-SNORA56 MUT2

3. Antibodies

GCLC (Western blot) Abcam ab207777

GCLC (IHC) proteintech 12601-1-AP

GAPDH proteintech 60004-1-Ig

Ki67 Abclonal A16919
