# Supplementary material for: SNORA56-mediated pseudouridylation of 28 S rRNA inhibits ferroptosis and promotes colorectal cancer proliferation by enhancing GCLC translation
Source: J Exp Clin Cancer Res. 2023 Dec 5;42:331. doi: 10.1186/s13046-023-02906-8 (PMC10696674; doi:10.1186/s13046-023-02906-8)
Supplement: Supplementary file 3 — Supplementary Material 3: Figs. 1–6.pdf. [file 13046_2023_2906_MOESM3_ESM.pdf]

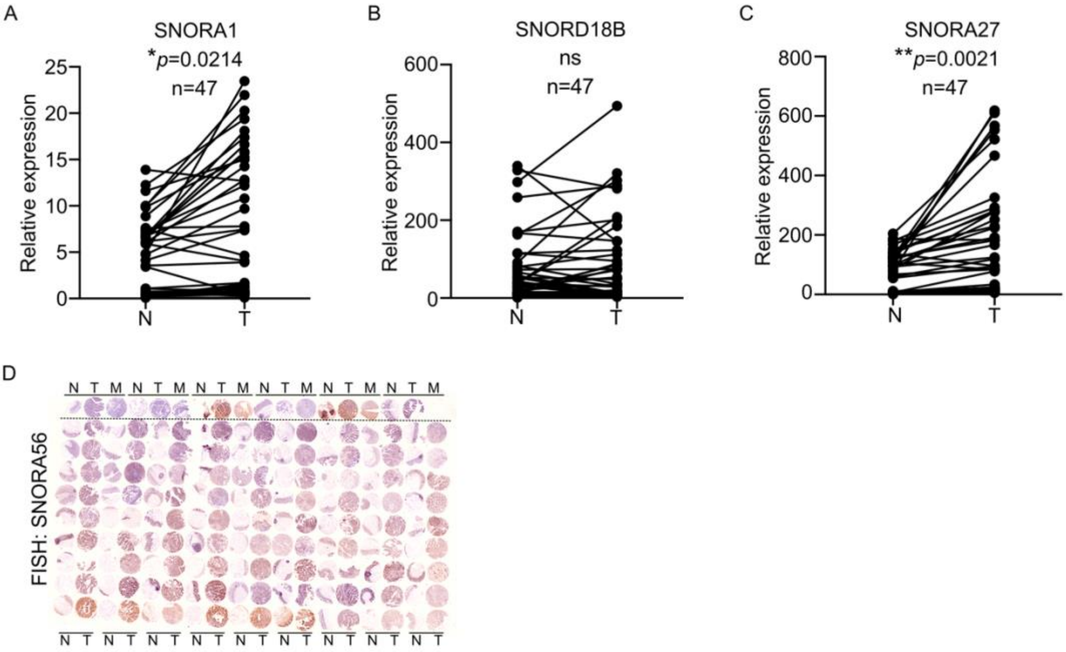

Figure.S1 Screening of the upregulated snoRNAs in CRC tissues and cells.

(A-C) Relative expression of SNORA1, SNORA27 and SNORD18B in 47 paired CRC and adjacent non-tumor tissues detected by qPCR. (D) The expression of SNORA56 examined by FISH using CRC tissue microarray.

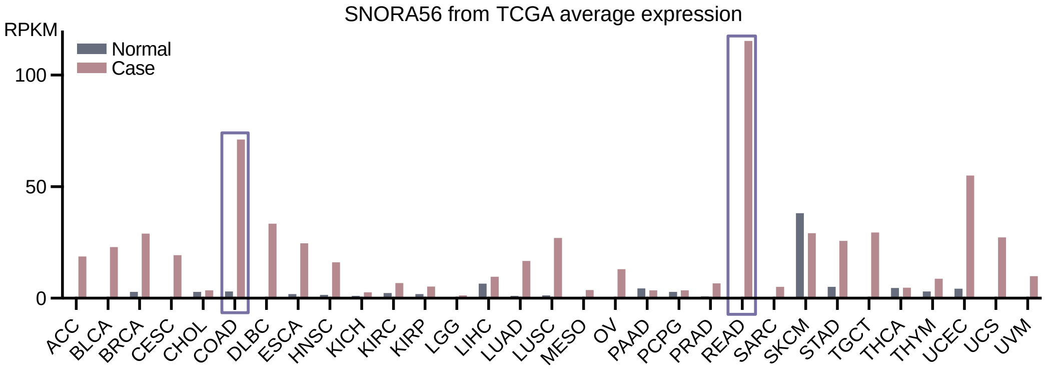

Figure.S2 Pan-cancer analysis of the SNORA56 expression.

Pan-cancer analysis on the average expression of SNORA56 from TCGA database.

RPKM, Reads Per Kilobase per Million mapped reads.

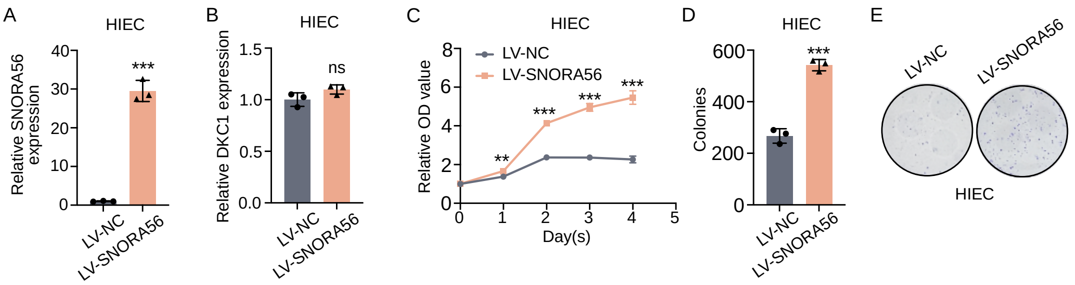

Figure. S3 SNORA56 accelerates the proliferation in HIEC cells. (A-B) Relative expression of SNORA56 and DKC1 measured by qPCR in HIEC cells stably transfected with LV-NC or LV-SNORA56 plasmids. (C) Proliferation ability detected by CCK8 in HIEC cells with SNORA56 overexpressed or its control. (D) Colony formation assay in HIEC cells with SNORA56 or empty vector plasmid transfection.

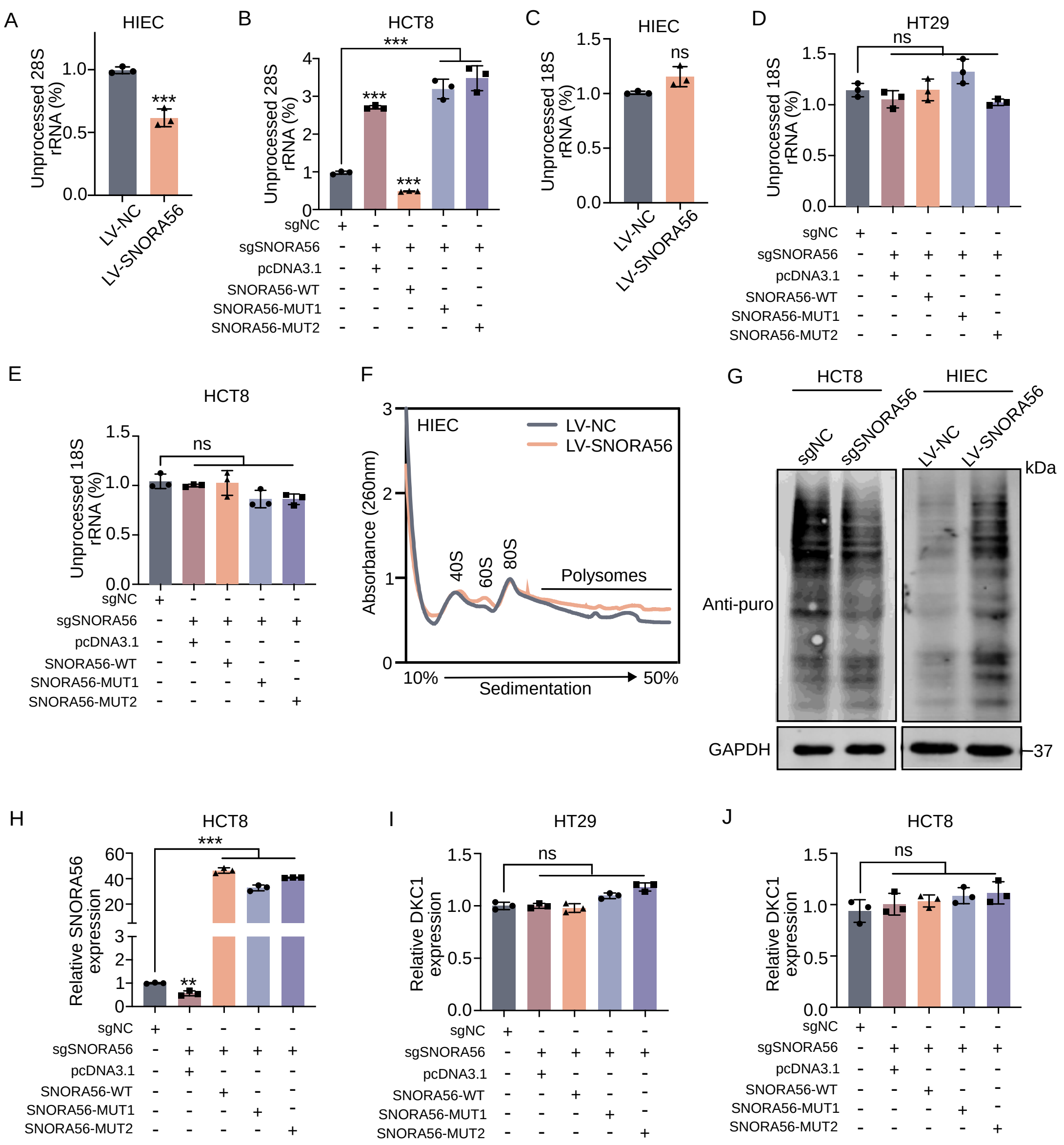

Figure. S4 SNORA56 promotes global translation via facilitating 28S rRNA maturation in CRC. (A) Relative unprocessed 28S rRNA rates detected by qPCR in HIEC cells stably transfected with SNORA56 or empty vector. (B) Relative unprocessed 28S rRNA rates detected by qPCR in HCT8 cells with indicated transfection. (C-E) Relative unprocessed 18S rRNA rates detected by qPCR in HIEC, HT29 and HCT8 cells with indicated transfection. (F) Polysome profiling assay of HIEC cells with SNORA56 or its empty vector overexpressed. (G) Western blot analysis using HCT and HIEC lysates with indicated transfection after O-Propargyl-

Puromycin (OP-puro) treatment. (H) Relative SNORA56 expression examined by qPCR in HCT8 cells with indicated transfection. (I-J) Relative DKC1 expression examined by qPCR in HT29 and HCT8 cells with indicated transfection.

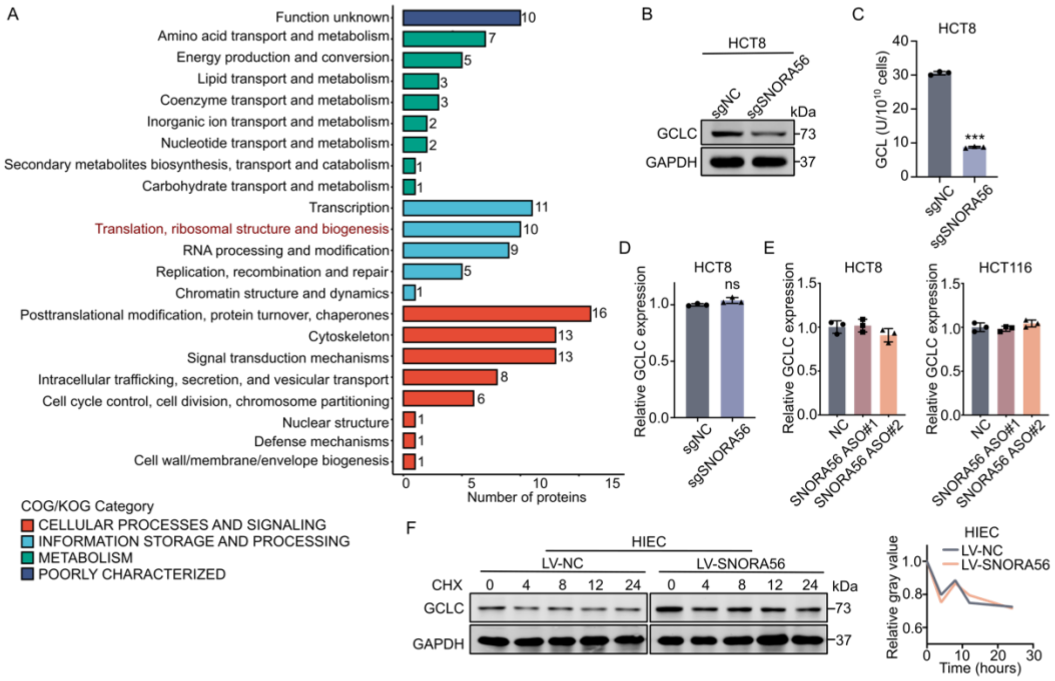

Figure. S5 SNORA56 regulates the translation of the downstream GCLC. (A) COG/KOG function classification of differential downregulated proteins upon proteomics. (B) Western blot analysis of the GCLC protein level in HCT8 cells with or without SNORA56 silencing. (C) The GCL enzyme activity in HCT8 cells transfected with sgNC or sgSNORA56. (D-E) Relative GCLC mRNA expression detected by qPCR in HCT8 and HCT116 cells with indicated transfection. (F) Protein stability assay of GCLC in HIEC cells with indicated transfection under CHX treatment for different times.

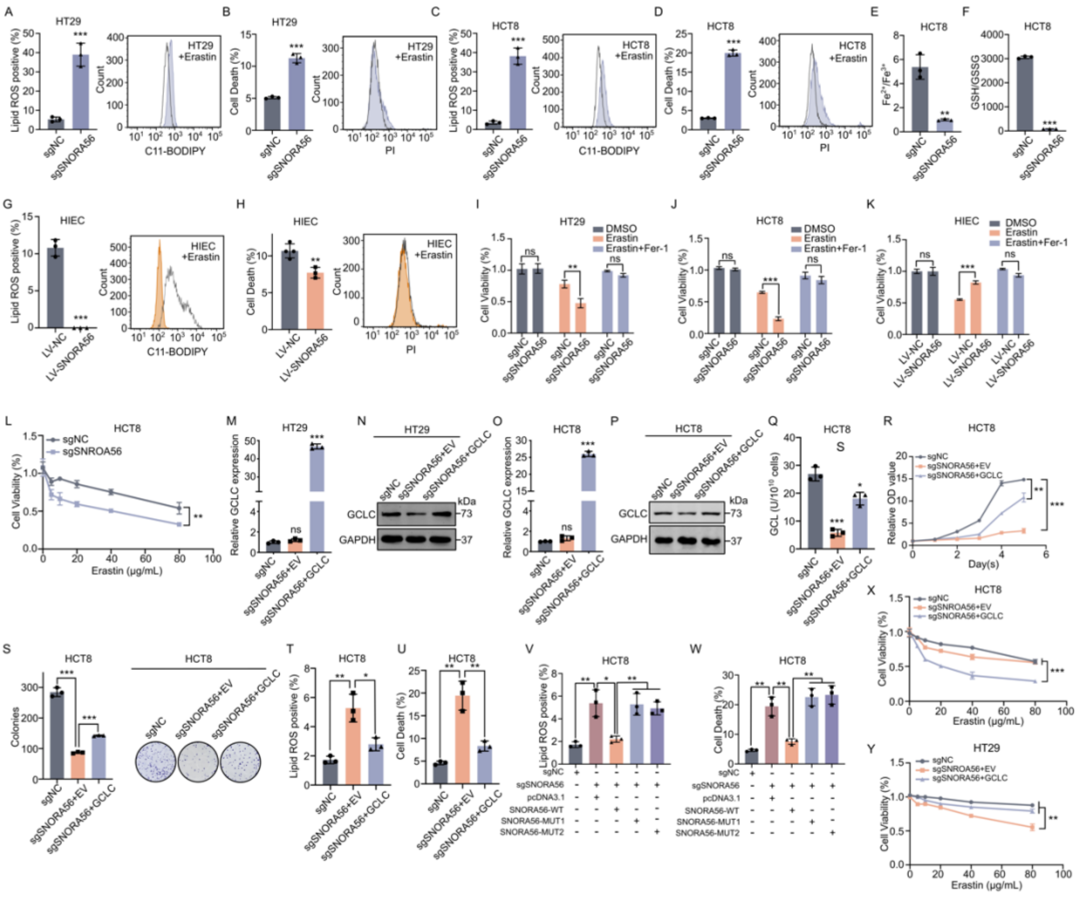

Figure. S6 SNORA56 stimulates proliferation and attenuates ferroptosis via GCLC in CRC. (A, C, G, T, V) Relative lipid ROS positive levels examined by flow cytometry using C11-BODIPY probes in HT29, HCT8 and HIEC cells with indicated stable transfection under erastin treatment. (B, D, H, U, W) Relative cell death rates measured by flow cytometry via PI staining in HT29, HCT8 and HIEC cells with indicated stable transfection under erastin treatment. (E) The content ratio of Fe^{2+} and Fe^{3+} in HCT8 cells transfected with sgNC or sgSNORA56 stably. (F) The content ratio of GSH and GSSG in HCT8 cells with SNORA56 stably knockdown or its control. (I-K) Cell viability of HT29, HCT8 and HIEC cells with indicated stable transfection detected by CCK8 under DMSO, Erastin and Erastin+Fer-1 treatment

respectively. (L, X-Y) The sensitivity of HCT8 and HT29 cells with indicated transfection to erastin at different concentrations. (M, O) Relative mRNA expression of GCLC detected by qPCR in HT29 and HCT8 cells transfected with GCLC overexpressed plasmids or empty vector (EV). (N, P) The expression of GCLC protein measured by western blot in treated HT29 and HCT8 cells. (Q) GCL enzyme activity of HCT8 cells stably transfected with sgNC, sgSNORA56, sgSNORA56 combined with GCLC overexpression plasmid. (R-S) Proliferation ability of HCT8 cells with indicated transfection using CCK8 and colony formation assay.
